# Vulnerability of populations and the urban health care systems to nuclear weapon attack – examples from four American cities

**DOI:** 10.1186/1476-072X-6-5

**Published:** 2007-02-28

**Authors:** William C Bell, Cham E Dallas

**Affiliations:** 1 Center for Mass Destruction Defense, College of Pharmacy, University of Georgia, Athens, GA 30602, USA

## Abstract

**Background:**

The threat posed by the use of weapons of mass destruction (WMD) within the United States has grown significantly in recent years, focusing attention on the medical and public health disaster capabilities of the nation in a large scale crisis. While the hundreds of thousands or millions of casualties resulting from a nuclear weapon would, in and of itself, overwhelm our current medical response capabilities, the response dilemma is further exacerbated in that these resources themselves would be significantly at risk. There are many limitations on the resources needed for mass casualty management, such as access to sufficient hospital beds including specialized beds for burn victims, respiration and supportive therapy, pharmaceutical intervention, and mass decontamination.

**Results:**

The effects of 20 kiloton and 550 kiloton nuclear detonations on high priority target cities are presented for New York City, Chicago, Washington D.C. and Atlanta. Thermal, blast and radiation effects are described, and affected populations are calculated using 2000 block level census data. Weapons of 100 Kts and up are primarily incendiary or radiation weapons, able to cause burns and start fires at distances greater than they can significantly damage buildings, and to poison populations through radiation injuries well downwind in the case of surface detonations. With weapons below 100 Kts, blast effects tend to be stronger than primary thermal effects from surface bursts. From the point of view of medical casualty treatment and administrative response, there is an ominous pattern where these fatalities and casualties geographically fall in relation to the location of hospital and administrative facilities. It is demonstrated that a staggering number of the main hospitals, trauma centers, and other medical assets are likely to be in the fatality plume, rendering them essentially inoperable in a crisis.

**Conclusion:**

Among the consequences of this outcome would be the probable loss of command-and-control, mass casualties that will have to be treated in an unorganized response by hospitals on the periphery, as well as other expected chaotic outcomes from inadequate administration in a crisis. Vigorous, creative, and accelerated training and coordination among the federal agencies tasked for WMD response, military resources, academic institutions, and local responders will be critical for large-scale WMD events involving mass casualties.

## Background

The increasing likelihood of the use of weapons of mass destruction (WMD) on large civilian populations has been described in international government alerts [1], U.S. Congressional hearings [2], research studies [3,4], and numerous scientific publications [5-9]. Islamic terrorist attacks on New York and Washington, D.C. have accentuated the reality of this threat, though the magnitude of casualties with WMD would be many times greater in scale. There is continued concern over the security of the enormous arsenal of nuclear, chemical, and biological agents left over in Russia as a result of the Cold War. It is known that Libya, Iran, Syria, Iraq, and North Korea have been actively recruiting the scientists that constructed this massive stockpile, and it is not certain where many of these experts are now [10].

While thousands of deaths occurred in a single day with the World Trade Center attack in New York, the impact on the health care system was not equivalently severe, as relatively few morbidity cases were produced. In most conceivable WMD attacks, however, it is reasonable to expect that the health care system would be overloaded with massive numbers of patients requiring an array of professionals with specialized training. If this already stretched medical community was also severely impacted by the very attack that requires its response, the effects would be even more devastating. In addition to the loss of medical care, among the anticipated outcomes for the general public will be fear of invisible agents and contagion, magical thinking about radiation, anger at perceived inadequacies by government entities, scapegoating, paranoia, social isolation, demoralization, and loss of faith in social institutions [11]. Intervention, guided by the appropriate use of WMD modeling software, would be the fastest and perhaps even the only effective means to effectively respond before loss of the sense of social and group responsibilities occurs, and before sufficient decline in the ideological metaphors which bind the community results in mass chaos and highly negative social sequelae.

### Integration of casualty estimates into current WMD response paradigms

Model estimates of casualty distributions could be of great benefit in mass casualty planning when utilized by the existing WMD response systems, though development of these approaches toward the extreme conditions of a nuclear attack are still underway. This process has advanced through a number of stages, with the magnitude of the response necessary for nuclear attack requiring extensive revisions. An early approach to linking first-responder, public health, and health care systems by the U.S. government was the Metropolitan Medical Response System (MMRS, originally known as Strike Teams) [12]. There were over 50 urban areas that developed MMRSs, which focus primarily on the training and coordination of local personnel within target communities.

Another initial approach was the upgrading of civilian first responders in 120 communities by the U.S. Army Soldier and Biological Chemical Command (SBCCOM). The Department of Justice's National Domestic Preparedness Office (NDPO), now part of the Department of Homeland Security (DHS), is currently processing information pertaining to law enforcement, emergency medical response, medical, and public health issues [13]. While there are various private efforts at planning a mass casualty response, the absence of a large-scale WMD experience has precluded validation of these efforts. Recently, the military has developed field deployable emergency response units, which could prove highly valuable in a WMD crisis if model predictions could target where they should be deployed. Health hazard surveillance, control, and the mitigation of effects in WMD incidents by mobile response units would be significantly enhanced by the availability of accurate mass casualty estimates by these kinds of model efforts. Deployment of these resources in a large scale crisis like nuclear detonation would be significantly enhanced by knowledge of the location of burn, trauma, and other casualties.

### Mass casualty estimation on a geographic basis

The Defense Threat Reduction Agency (DTRA) has expended considerable effort to develop models for calculating mass casualties from a nuclear detonation. In order to specifically evaluate urban medical systems vulnerability we are employing the PC based Consequence Assessment Tool Set (CATS) v6, with ESRI's ArcGIS9 [14], CATS/JACE (Joint Assessment of Catastrophic Events) v5 with ESRI's ArcView 3.3, Hazard Prediction and Assessment Capability (HPAC) V4.04SP3 [15], as well as custom GIS and database software applications. HPAC does excellent Chemical Biological and Nuclear (CBN) modeling, although output could provide more flexibility. Additionally, results can be exported to CATS for further analysis and display. All three programs can access the current weather data from both classified and unclassified weather servers. Examples of uses of CATS/HPAC are hurricane, tidal surge and earthquake damage, prediction of the results from nuclear, biological and chemical releases, assessment of persons and infrastructure affected and at risk (e.g. which hospitals and pharmacies are under a CBN plume and are thus out of commission), and mobilization of surviving and nearby infrastructure outside the plume that would be needed to address healthcare and other emergency response needs of the community.

These models have been, and continue to be, developed with a view to better estimating the impact of WMD weapons in an offensive setting. However, recent DTRA enhancements and our modifications have facilitated their use in helping estimate potential casualties from a WMD terrorist incident. One area of intense interest, and somewhat of a vacuum in public health planning, has been the utility of this approach in estimating medical care vulnerabilities in such an attack, and for the calculation of the distribution of surviving medical care resources. While much work has already occurred in estimating the impact of chemical weapons (due to the dual use in chemical spill management from transportation and industrial accidents), or in nuclear power plant accident management, much less research and development has gone into estimating the impact on our civilian population of a nuclear weapon detonation from a terrorist incident in a large urban area. The models already calculate such factors as the impact of blast, thermal effects and fallout, but results are often not available at the detail level needed for civil defense purposes, casualty management, and planning the use of scarce health resources in response to a nuclear weapon detonation. Furthermore, the models do not readily facilitate the calculation of injuries from multiple effects such as burns and blast with fallout or prompt radiation. The complexity of the urban three-dimensional landscape and its local impact on thermal, blast and radiation is also poorly understood. Additionally, given their traditional world-wide focus, and the increased sensitivity to providing information on the U.S.A. of use to terrorists, the models do not provide detailed or current data that exists for the United States that would help provide better casualty estimates and response. The models can be customized locally and data updated if the user has sufficient expertise. However, there is often a significant duplication of effort due to overlapping jurisdictions and the lack of data sharing due to security and other considerations.

CATS and HPAC are also useful for creating realistic scenarios for training and planning before a disaster strikes, thus enabling responders to drill and exercise so they know roughly what to expect and how to react. Contingency plans can be created using comprehensive national and more detailed population and infrastructure data. Should disaster strike, the affected population and the impact on critical facilities can be quickly assessed, although efforts frequently need to be expended to ensure regional and local databases are current and useful.

### Utilization of model casualty estimates in medical planning

Without the directed use of accurate casualty distribution estimates, it is likely that past failures in mass casualty planning in large-scale medical disasters will be repeated. During the Sarin attack on Tokyo, hospitals became part of the problem when 23% of the healthcare workers became ill by unintentionally spreading the nerve agent to hospital and emergency staff workers. During the SARS epidemic in China, hospitals in Beijing and Hong Kong became "Super Seeders" of the *cornavirus *and dramatically accelerated contagion up to 250 individuals per day. A study by the American College of Emergency Physicians (ACEP) Task Force found that "little or no WMD-based expertise" existed among medical staff workers in hospitals [16].

Based on information from the National Commission on Terrorist Attacks upon the United States (9–11 Commission)[17], public hearings on the initial response show a terrible confusion among first responders that resulted in the addition of a "Catastrophic Incident Annex" to the second draft version of the National Response Plan (NRP) [18]. First responders during 9/11 suffered from an inability to communicate information concerning the scale and magnitude of the disaster, and thereby released conflicting public service information during the crisis that resulted in additional loss of life. The findings of these hearings show a critical need for a "National Strategy" for medical response to catastrophic incidents. The requirements of the Catastrophic Incident Annex exceed the CDC and HRSA benchmarks of 500 hospital beds for a population of one million needed for natural disasters.

For an effective response, delineating the geographic zones in which different types of injuries are likely to be found, and delineating zones in which victims are likely to sustain multiple injuries, is critical. In the case of a nuclear explosion, thermal effects will produce very large numbers of burn casualties – a dramatic medical and security challenge that differs from routine medical emergencies or non-nuclear WMD events. Multiple trauma injuries will accompany the injuries inflicted by thermal radiation. These will be qualitatively similar to current trauma protocols, with the exception of fallout contamination, but will differ drastically on the quantitative level. Additionally, certain regions will experience the unique casualties from prompt and fallout radiation. Multiple effects make for sicker patients, slower recoveries, and greater danger of severe sickness or death, especially among the old, the young, and the infirm.

A future goal for this work will be to focus on identifying those geographic areas and those combinations of casualties for which scarce medical resources can do the most good in the early stages of a disaster. This will help commanders determine where, among the harder hit areas, they should turn their attention as more resources come to bear. Currently, casualty management modeling and resource estimation support tools such as NBC CREST [19] exist for the military, but much work needs to be done to modify them from a military focus and make them useful in a civil defense environment. Work also needs to be done on identifying zones of different types of multiple injuries and estimating the impact of a fleeing population on casualties requiring treatment in various zones.

### Use of model estimations to help address limitations in mass casualty resources

Once accurate model estimations of mass casualty distributions are available, this data could be invaluable in the distribution of limited medical response resources in a WMD crisis in order to minimize mortality and morbidity in mass casualties. Although the National Disaster Medical System has voluntary access to 100,000 hospital beds nationwide, getting patients to these widely dispersed beds in time would be a logistical nightmare in nuclear as well as other WMD scenarios. A particularly dangerous deficiency is the lack of equipment for patient respiration and supportive therapy nationwide [20]. In a crisis in which there are tens of thousands of victims requiring respirators, there is certainly a potential for most of the more critical cases to perish. An ironic feature of the recent terrorist attacks in New York was the lack of impact on the health care system there, since most of the victims in the World Trade Center collapse died, without producing large numbers of ancillary casualties. However, nuclear detonation, as well as most WMD attacks, would be expected to produce the need for large mass casualty resources, including respirators. A national pharmaceutical stockpile has been created by the Centers for Disease Control to provide large supplies of many of the pharmaceutical agents that we would expect to need in likely WMD attack scenarios [21]. Arrangements are in place to use commercial carriers to speed elements of the stockpile to the various locations in which the attacks occur. Selection of the locations to place these critical distribution points would be considerably expedited by accurate predictions of where the casualties that critically need them would most likely be located.

## Data and methods

### Study area

Four of the top ten sized cities (New York City, Chicago, Washington, D.C. and Atlanta) were selected for this study of the impact of downtown nuclear detonations on populations and health care systems. All four cities are considered potentially high risk cities for a terrorist event.

### Size of weapon

Two sizes of nuclear weapon were simulated. The explosion of a tactical nuclear weapon with a predicted yield of 20 kilotons (Kt) and the explosion of the most common size of strategic weapon in the Russian arsenal with a 550 Kt yield. A fission fraction of 1 was assumed for the smaller device and 0.8 was assumed for the 550 Kt device [22]. Both weapons were assumed to explode close to the ground surface, as in a truck or a ship. Bursts at higher levels would cause greater thermal and blast effects which would be somewhat offset by lower downwind radiation amounts.

### Affected population

Population calculations were based upon block level data from the 2000 census, so calculations are based upon night time population data. In downtown areas, daytime populations, and therefore casualties, would be higher. Secondary deaths from radioactive fallout and other effects of the blasts would greatly increase the immediate deaths. Daytime building population estimates are rarely available but can be very high. Some examples of daytime populations for individual buildings are: Illinois Center in Chicago – 40,000; Empire State Building, New York – 20,000; former World Trade Center Complex – 50,000 employees with up to 100,000 visitors daily. The total nighttime population in Manhattan is roughly 1.5 million rising to 2.1 million with workers during a typical day. To this number must be added visitors for special events and tourists, a number that is highly variable, and for which no official estimates exist [23]. For Washington, D.C. Homeland Security Council (HSC) [24] quotes Oak Ridge National Laboratory's (ORNL) estimate of the daytime population at 1,066,666 and the nighttime population at 571,476, yielding 495,190 additional people during the day. HSC estimated an additional 701,000 people by day within 11 kilometers (kms) of downtown Washington of which 481,000 were within a 5 km radius of downtown, and an additional 220,000 were distributed in a donut shape with an outer radius of 11 kms and an inner radius of 5 kms. In the absence of building level data, the National Planning Scenarios suggest a better estimate of daytime population for Washington can be obtained by adding an additional 6124 people per square km to the 5 km central part of Washington and 579 people per square km to the 5–11 km ring, [24]. In the case of Manhattan, spreading the additional 600,000 daytime population evenly adds an additional 7,059 per square km during daytime, giving a better approximation of 24,706 per square km for the daytime population without visitors.

### Hospital data

Data on the number and types of hospital beds were obtained from DTRA's CATS/JACE database and updated from ESRI's 2004 Data and Maps [25] and InfoSource's American Directory of Hospitals 2004 [26]. Psychiatric and other special hospitals were removed from consideration. Some discrepancies were fixed using the American Hospital Directory [27].

### Weather and climate data

Weather and climate has a significant effect on impacts resulting from a nuclear detonation [28]. Wind is one major factor, as wind carries the resultant fallout cloud downwind. Atmospheric stability affects the height of the typical mushroom cloud and behavior of the fallout plume, and the amount, thickness and height of clouds impact the scattering, reflection and absorption of radiation. Detonations occurring below clouds have a much greater impact on thermal radiation as radiation is reflected back down to earth, while detonations above cloud reflect radiation out to space and reduce radiation at the surface. Snow also enhances the effect of thermal radiation through its high albedo. Snow and cloud together typically increase the impact of thermal radiation roughly twofold, but in extreme situations, with high visibility beneath dense clouds, there can be up to five times the radiation of a clear day [29].

Average upper air climate for a month or season does not estimate nuclear effects well, as averaging a north and south wind could cancel each other out. What is needed is a synoptic climatology of typical upper air conditions for major cities. These data are currently not available, so for this study we selected a number of case studies from upper-air radiosonde data for particular days, and we have used these weather conditions as model inputs. The data were selected after looking at three years of twice daily skew-T Log P thermodynamic diagrams from the Plymouth State University's meteorology program WEB site to get a better understanding for the data [30]. The days we have selected are significant days when winds generally ran in a direction with major impact on the health care system. However, these days are not isolated, as similar patterns were seen to repeat on many other days. When analyzing data for civil defense purposes it is critically important not to underestimate the potential impacts of the catastrophe being analyzed. For our selected case studies, data was input on pressure, altitude, temperature, wind speed, wind direction and humidity for several levels up to the 300 millibar level or about 9,000 meters (m).

### Model used and sources of uncertainty

We used the DTRA's CATS-JACE model to simulate the effect of fallout radiation from a nuclear explosion [31], EM-1 to calculate blast effects [32], and Brode's work [33], as modified by Binninger [34] to calculate thermal fluence, using thermal fractions as discussed in Northop [35]. With any such models there are many sources of uncertainty in the input parameters which can be expected to impact the accuracy of the predictions.

### Atmospheric effects

As noted above, atmospheric conditions affect the quantity of energy absorbed, reflected and scattered, with a highly significant impact on casualty distributions. Near surface bursts create craters and large amounts of dust and solids from the ground, or buildings are thrown into the air. Low cloud above the fireball will cause a considerable degree of reflection back to the surface which will reflect from many different angles and considerably increase the impact of thermal radiation and favor mass fires. Fresh snow on the ground would also reflect the radiation, further increasing the thermal impact.

Wind speed and direction have a tremendous impact on where fallout radiation is deposited. This depends upon many factors, from the overall synoptic situation and topography to local turbulence and surface roughness, land use, and street width and orientation. Models give better results when current three dimensional weather data are utilized as input, along with detailed topography and land use. However, generally speaking, much further work needs to be done before dispersion models can provide detailed, realistic results in complex city centers. Observers have noted large changes in radiation fallout over small distances caused by variations in local atmospheric conditions and topography [36].

### Protection offered by buildings and vehicles

Buildings provide various degrees of protection from radiation according to the type of construction and location. The level of protection offered typically varies between 10% and 80%. Some of the factors which affect protection include whether the building is in an urban or rural area, the roof and wall type and thickness, number of floors and location of office or home relative to other floors, e.g., single story, multistory, basement, top floors, middle floors and lower floors and whether glass is shattered by blast [37,38]. Blast damage greatly reduces the protection factors through the blowing in of doors, loss of roof integrity, and breaking of windows. At Hiroshima, windows were broken at a radius of 15 kms by overpressures of only a fraction of a pound per square inch and in exceptional cases were broken up to 27 kms away [38]. Using typical figures from Hiroshima and the cube law for blast extrapolation, one could expect windows to break at up to 17.5 kms for 20 Kt and 53 kms for 550 Kt detonations. Injury thresholds for window glass are considered to be about 0.6 pounds per square inch (psi) [26] or 6 kms for 20 Kt and 18 kms for 550 Kt detonations from fig 2.29 [34]. Recent research [39-41] has shown that buildings, even in their best condition, fail to provide good filtration from radioactive particles in the 1–10 micron range, where the greatest health threat exists.

The highest impacts of radiation generally occur when people are caught in the open, or, are tied up in traffic jams trying to escape in vehicles, which provide little protection against fallout. Based on evidence from recent natural disasters in Louisiana and Florida it is likely that major exit arteries after a nuclear event will be completely impassable during the time period when fallout is at a maximum, exposing fleeing population to high levels of fallout. It is also expected that due to lack of information getting to the public, many people will try to flee by car or on foot, often in the wrong direction, again exposing themselves to high levels of radiation, as vehicles provide virtually no protection. Shelter-in-place options are poorly understood, and without effective communications and well thought out and prepared plans by both authorities and potential victims, could prove equally disastrous.

Buildings also protect against thermal effects by blocking a direct line of sight to the detonation. Thermal effects may be affected by such factors as the number, size and orientation of windows; presence or absence of intact windows after the blast; size, number of panes and tinting of glass, presence or absence of bug screens, and height, spacing and orientation of buildings. Window coverings and type of furniture and furnishings will respond differently to the increased thermal surge, with some materials being more susceptible to burning than others.

## Discussion and results

### Effects of nuclear weapon detonations

#### Thermal effects – fires and burns

**Figure 1:**
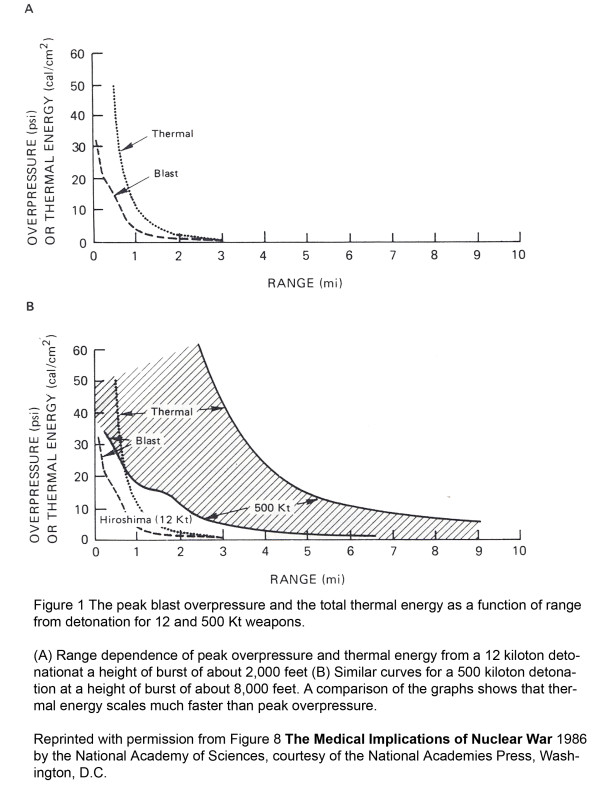
**Peak blast overpressure and total thermal energy as a function of range from detonation for 12 and 500 Kt weapons**. **(A) **Range dependence of peak overpressure and thermal energy from a 12 kiloton detonation at a height of burst of about 2,000 feet. **(B) **Similar curves for a 500 kiloton detonation at a height of burst of about 8,000 feet. A comparison of the graphs shows that thermal energy scales much faster than peak overpressure. Reprinted with permission from Figure 8 **The Medical Implications of Nuclear War **1986 by the National Academy of Sciences, courtesy of the National Academies Press, Washington, D.C.

**Figure 2:**
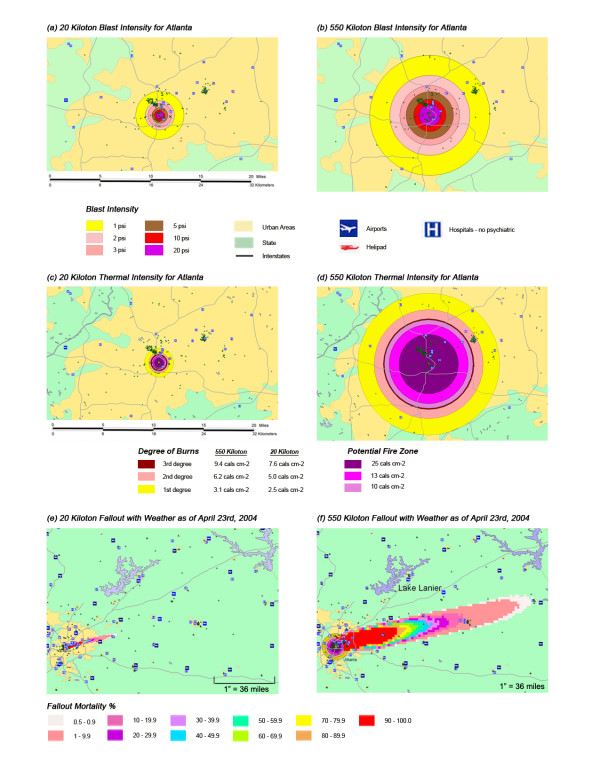
**Blast, Thermal and Fallout Effects for 20 Kt and 550 Kt Nuclear Explosions in Atlanta**. **(a) **20 Kt Blast Intensity. **(b) **550 Kt Blast Intensity. **(c) **20 Kt Thermal Intensity, **(d) **550 Kt Thermal Intensity. **(e) **20 Kt Fallout, **(f) **550 Kt Fallout.

The thermal impacts of a nuclear explosion are always large but scale much faster than blast with larger yield detonations. Thermal radiation decays as the inverse square of the distance from the detonation, while blast decays as the inverse cube of the distance. Figure 1[42] shows the blast and thermal effects from a low free air burst for a 12 Kt (Hiroshima size) and a 500 Kt typical Russian warhead It shows the much larger rate of increase of the thermal component compared to the blast component in going from the 12 Kt to the 500 Kt devices. A similar effect for 20 and 550 kiloton devices is shown in Figures 2a through 2d, using Atlanta as an illustration. For large weapon sizes (> 100 Kt), significant thermal effects extend to much greater radii than substantial blast effects.

Absorption of thermal energy can cause fires in the vicinity of the detonation point and burns to individuals, either directly from flash burns or indirectly from the mass fires themselves. Binninger et al [34] have conducted work for DTRA on fire prediction modeling. In urban environments, a large number of variables can affect the intensity and impact of the thermal pulse. These include the weapon yield, the fraction of the total yield emitted as thermal radiation, the distance between the weapon and point of interest, and the thermal radiation transmissivity through the immediate atmosphere. EM-1 [32], Brode [33], Binninger [34], Northop [35] and Glasstone [37] have all calculated the thermal fluence for any point at a distance from ground zero, and we have used Brode's method, as modified in Binninger.

Clouds and the presence of snow have a major impact, and, as noted above, if the fireball occurs below thick continuous cloud, a five-fold increase in reflection may occur. Recent snow cover further increases effects, although the study of cloud and snow interaction is a subject for further research. Cloud height, thickness, type, atmospheric scattering, dust particles in the air, humidity, building orientation and size and location of windows, all have effects, as do type and quantity of flammable materials that will be illuminated within a room. Building construction also plays a major role in room to room and floor to floor spread, as do separation and orientation of neighboring distances [42].

In general, fire effects of nuclear weapons are not as well developed as the modeling of blast and target destruction, yet it is recognized that casualties resulting from fires, and burns in nuclear attack would be of major impact for civil defense [43] and emergency health care. Major fires can occur when thermal fluences exceed 10 calories (cals)/cm^2 ^and are very common with fluences over 25 cals/cm^2^, although this varies with the type of construction, building contents, and morphology of the city [34,43]. Fires will start much easier when windows are blown out as glass greatly reduces the thermal fluence inside a room. Skin burns are generally classified into first (like very bad sunburn), second (produce blisters that lead to infection if untreated and permanent scars) and third degree burns (which destroy skin and underlying tissue) and are dependent upon the intensity of the radiant exposure and the size of the explosive device (Table 1 from Fig 12.65 [37]). The entire US has specialized facilities to treat roughly 1,500 burn victims, which is far less than the burn casualties produced by one single small nuclear explosion. Additionally, most of these beds are already occupied.

**Table 1:** Intensity of thermal fluence by thermal effect type

Type of Thermal Effect	20 Kt Device Thermal Fluence Calories/cm2	550 Kt Device Thermal Fluence Calories/cm2
Mass Fires Virtually Certain	25	25
Mass Fires Likely	13	13
Mass Fires Possible	10	10
3^rd ^Degree Burns (50% chance)	7.6	9.4
2^nd ^Degree Burns (50% chance)	5.0	6.2
1st Degree Burns (50% chance)	2.5	3.1

The thermal effects listed in tables 2 and 3 refer to block level Census 2000 or nighttime affected population that are within the given thermal contour. For populations within the mass fire contour (13 cals/cm^2^) very few people will escape without some form of significant injury. In the third degree burn zone there will be many burns from resulting fires as well as those directly affected by flash burns from the detonation. For the first and second degree burn zones, the number of people exposed (i.e. in direct line of sight to the fireball) will vary greatly by time of day, time of year, weather, city and building morphology. Weather factors such as cloud above the fireball and snow on the ground will aid in multiple and omni-directional reflection of radiation and greatly increase the numbers and average intensity of burns. Typical exposure proportions of affected population actually receiving first, second or third degree burns will range from 1% to 25% of those affected [44,45], with the recent National Planning Scenarios assuming 15% as a reasonable average [[26], page I-28]. For a spring/early summer day at lunchtime or on a weekend afternoon, 25% would not be unreasonable.

**Table 2:** Affected populations from 550 Kt surface detonations in 4 downtowns

**Effect Type**	**City**			
	Washington	New York	Chicago	Atlanta
Combined Fallout and Thermal	2,678,638	6,456,056	3,398,527	1,243,165
Combined Fallout and Blast	2,541,368	6,001,862	3,167,676	1,178,751
All Thermal categories	923,401	3,309,930	1,614,371	459,639
All Fallout categories	2,170,917	5,042,904	2,430,731	1,064,928
All Blast categories	708,710	2,554,308	1,251,965	353,925
				
All Thermal categories	923,401	3,309,930	1,614,371	459,639
> 25 cal cm^2 ^Zone	211,206	903,591	316,847	122,572
> 13–25 cal cm^2 ^Zone	135,752	521,519	305,725	68,720
3rd Degree Burn Zone	94,202	315,388	190,071	42,141
2nd Degree Burn Zone*	165,557	509,926	256,134	71,496
1^st ^Degree Burn Zone	316,684	1,059,506	545,594	154,710
				
All Fallout Categories where mortality > 0.5%	2,170,917	5,042,904	2,430,731	1,064,928
Mortality > 90%	1,016,206	3,229,502	1,473,337	614,767
> 50–90%	583,486	493,519	261,381	123,160
> 10–50%	311,292	678,783	180,456	119,567
> 0.5–10%	259,933	641,100	515,557	207,568
				
All Blast categories over 1psi	708,710	2,554,308	1,251,965	353,925
> 20psi	20,710	158,889	52,950	19,476
10–20psi	32,703	155,019	34,704	25,437
3–10psi	158,287	596,150	231,341	77,985
2–3psi *	138,363	537,279	316,306	71,105
1–2psi	358,647	1,106,971	616,664	159,922

• Approximately 500,000 additional daytime workers will be affected for Washington and 600,000 for New York for the second degree burn zones or the 2psi zone. No data are available for Atlanta and Chicago.

**Table 3:** Affected population from 20 Kt surface detonation in 4 downtowns

**Effect Type**	**City**			
	Washington	New York	Chicago	Atlanta
Combined Fallout and Thermal	188,430	1,649,587	614,535	182,717
Combined Fallout and Blast	223,570	1,733,983	637,033	207,025
All Thermal categories	39,641	140,701	79,451	36,256
All Fallout categories	172,819	1,592,968	554,048	160,224
All Blast categories	92,040	286,587	104,988	63,814
				
All Thermal categories	39,641	140,701	79,451	36,256
> 25 cal cm^2 ^Zone	1,024	12,336	11,574	1,431
> 13–25 cal cm^2 ^Zone	963	15,303	14,685	1,810
3rd Degree Burn Zone*	3,132	20,660	12,456	5,001
2nd Degree Burn Zone**	9,876	22,993	10,381	8,593
1^st ^Degree Burn Zone	24,646	69,409	30355	19,421
				
All Fallout Categories where mortality > 0.5%	172,819	1,592,968	554,048	160,224
Mortality > 90%	80,386	429,172	252,538	46,579
> 50–90%	14,851	145,123	52,182	11,632
> 10–50%	28,335	358,922	85,807	43,970
> 0.5–10%	49,247	661,177	163,521	58,043
				
All Blast categories over 1psi	92,040	286,587	104,988	63,814
> 20psi	902	8,616	4,222	961
10–20psi	658	10,056	9,799	1,342
3–10psi	9,194	46,515	33,410	12,949
2–3psi ***	22,532	55,126	23,360	15,278
1–2psi	58,754	166,274	34,197	33,284

To take into account daytime populations near central business districts [26]

* Add roughly 32–35,000 additional people within 3rd degree burn isoline

** Add roughly 53–60,000 additional people within 2nd degree burn isoline

*** Add roughly 50,000 additional people within the 3psi isoline and 90,000 within the 2psi isoline.

**Figure 3:**
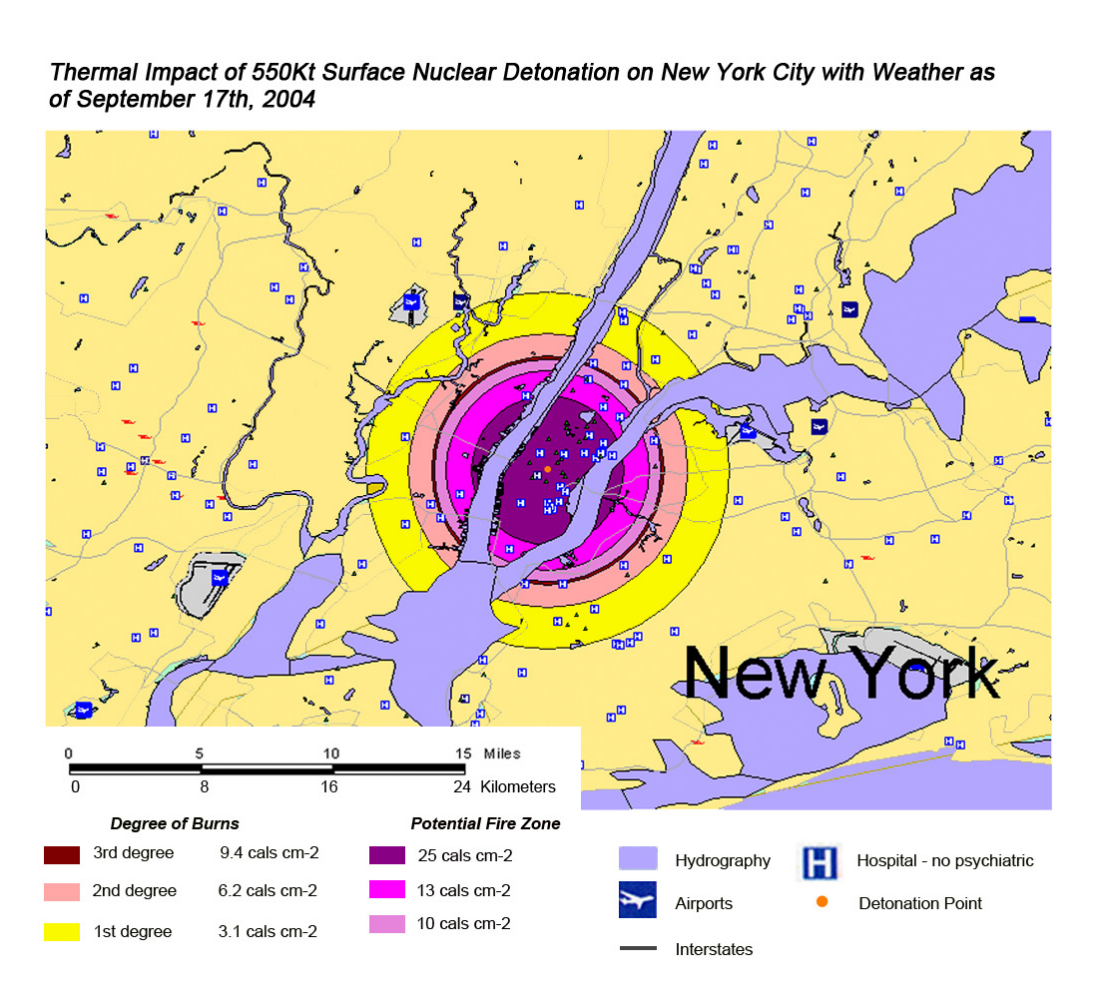
Thermal Impact of 550 Kt Surface Nuclear Detonation on New York City with Weather as of September 17^th^, 2004.

**Figure 4:**
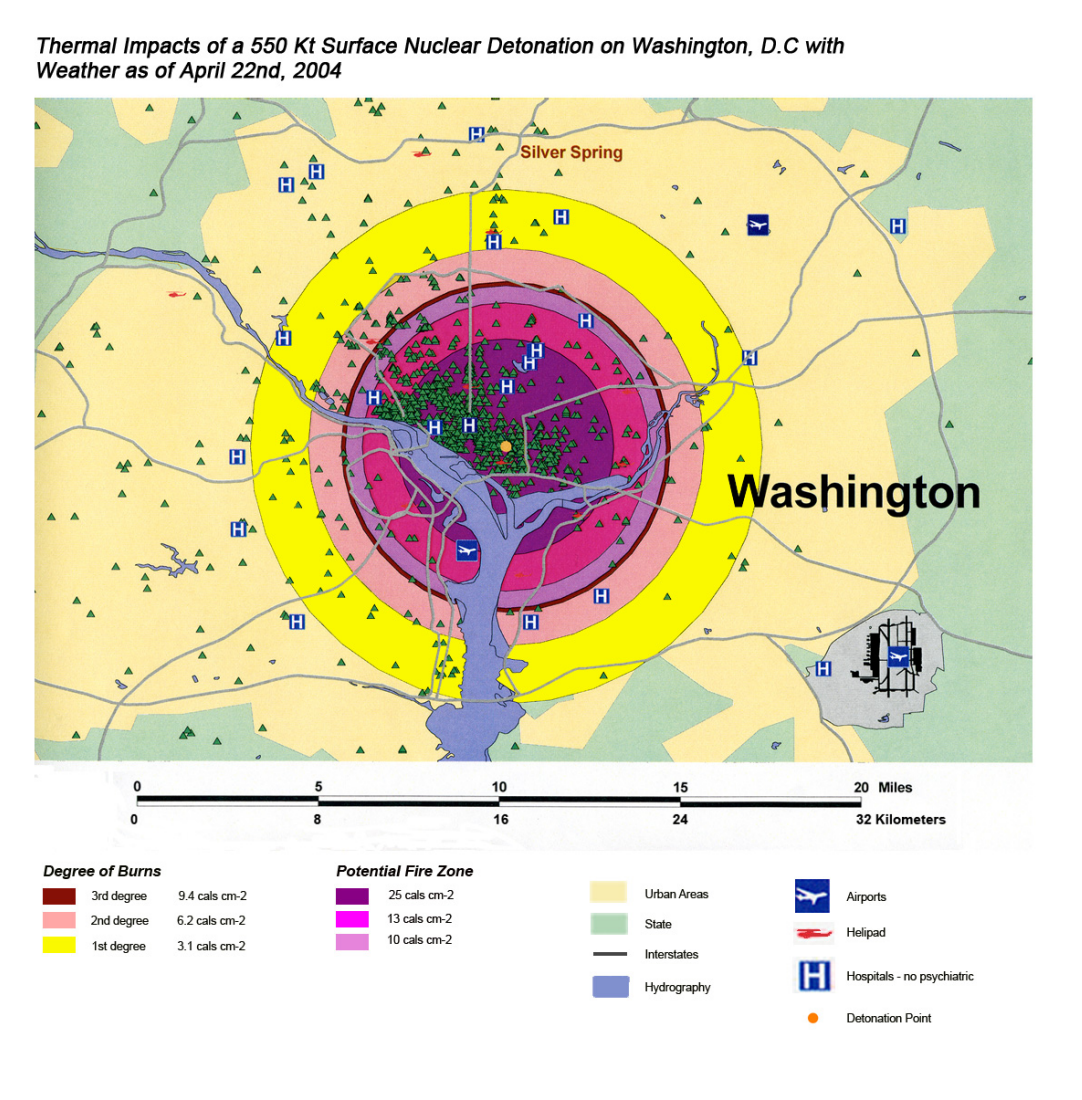
Thermal Impact of a 550 Kt Surface Nuclear Detonation on Washington, D.C. with Weather as of April 22^nd^, 2004.

**Figure 5:**
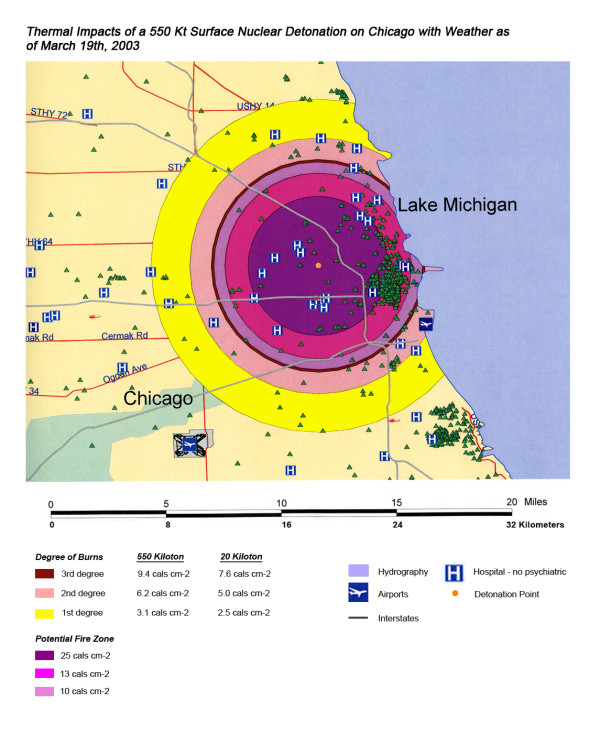
Thermal Impact of a 550 Kt Surface Nuclear Detonation on Chicago with Weather as of March 19^th^, 2003.

**Figure 6:**
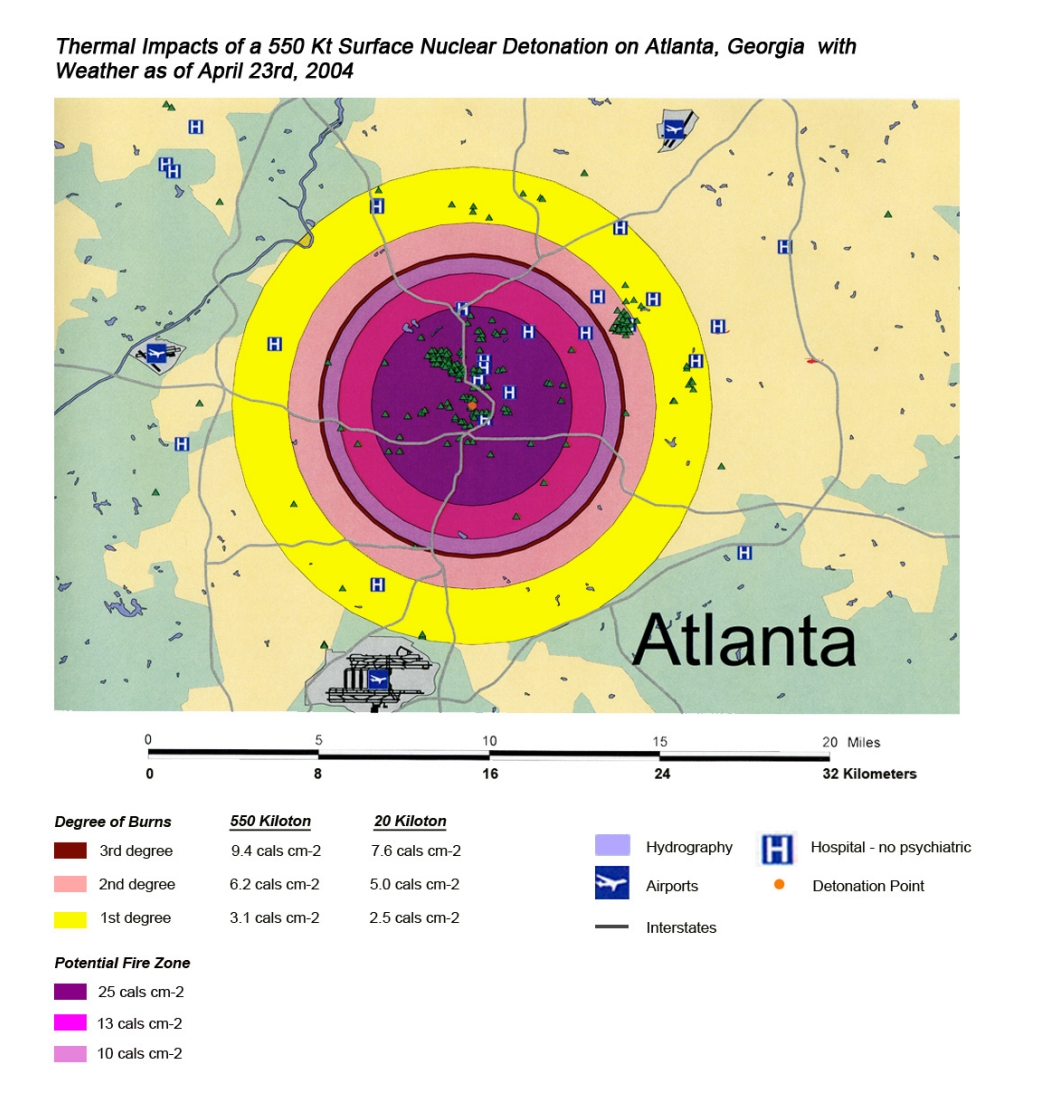
Thermal Impact of a 550 Kt Surface Nuclear Detonation on Atlanta with Weather as of April 23^rd^, 2004.

The areas of New York, Washington, D.C., Chicago and Atlanta affected by thermal radiation from a 550 KT nuclear detonation are shown in Figures 3, 4, 5, 6 respectively. The destruction of the major hospitals in the downtown areas is nearly complete in all four cities. Hydrology of the urban areas can be a significant factor with the impact of rivers, lakes, and ocean systems in and adjacent to the urban areas. In New York (Figure 3), with the division of Manhattan from the other city areas by two rivers emptying into the ocean, the loss of the hospital infrastructure is alleviated somewhat by the wider geographic distribution of the health care system (the dense urban packing of hospitals still intensifies the hospital bed loss). In Washington D.C. (Figure 4), hospital distribution occurs primarily north of the Potomac River, further concentrating urban health care systems in the areas significantly impacted by the severe zones of thermal effect. Location next to a large body of water, like Chicago adjacent to Lake Michigan (Figure 5), tends to dissipate much of the thermal damage over water when ground zero is in the downtown area. Inland cities like Atlanta (Figure 6) do not have these mitigating factors, and hospital distribution follows primarily economic factors.

#### Blast damage

Most damage to buildings in cities comes from explosive blast. The blast drives air away from the explosion causing objects to be crushed and high winds that can knock objects down, such as people or trees. Four pounds per square inch (4psi) is usually enough to destroy most residential dwellings. Most blast deaths occur from the collapse of occupied buildings, or from people being blown into objects, or objects impacting people. Typically, about half the people whose low rise buildings collapse on them survive the collapse.

**Figure 7:**
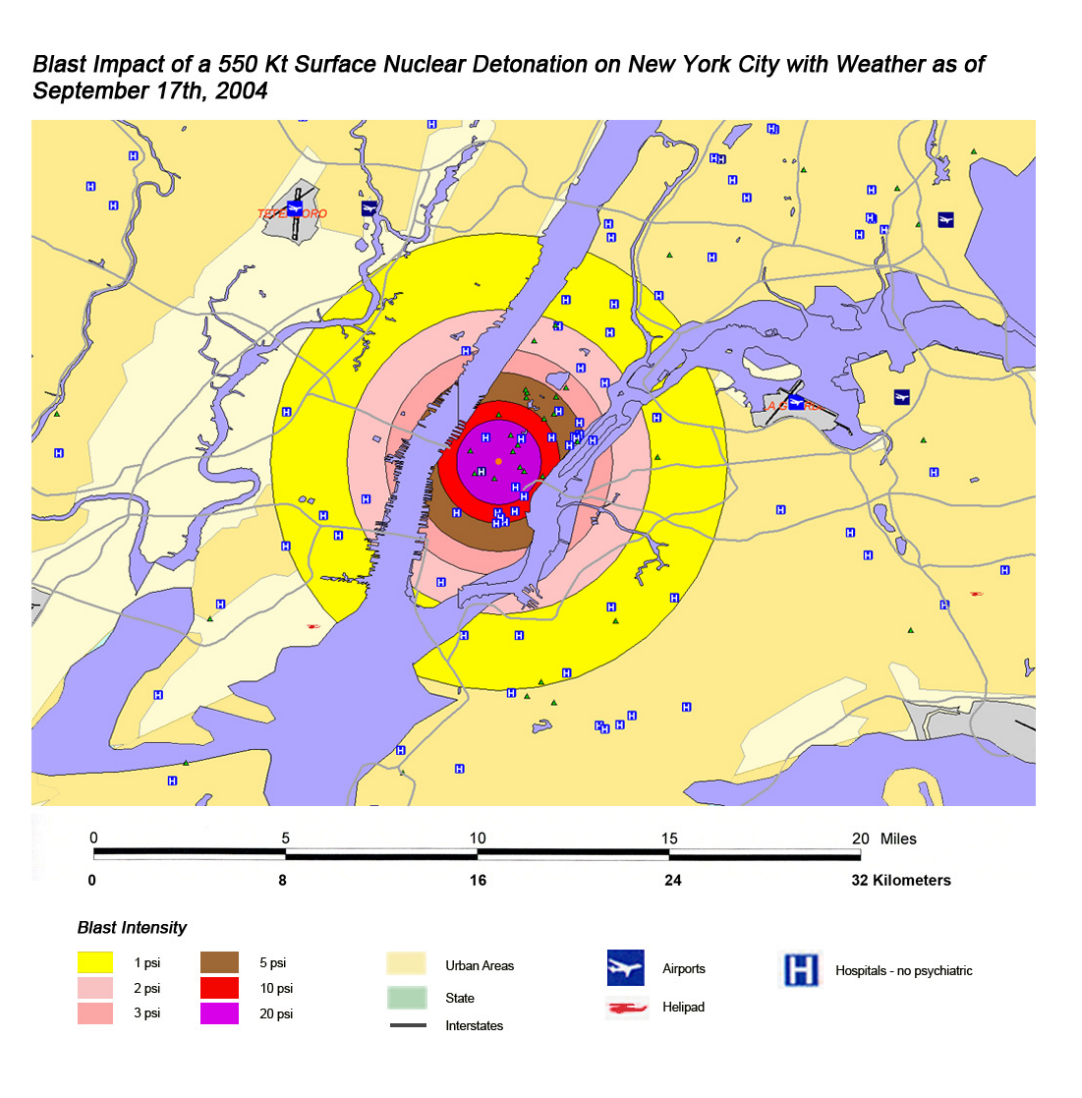
Blast Impact of 550 Kt Surface Nuclear Detonation on New York City with Weather as of September 17^th^, 2004.

**Figure 8:**
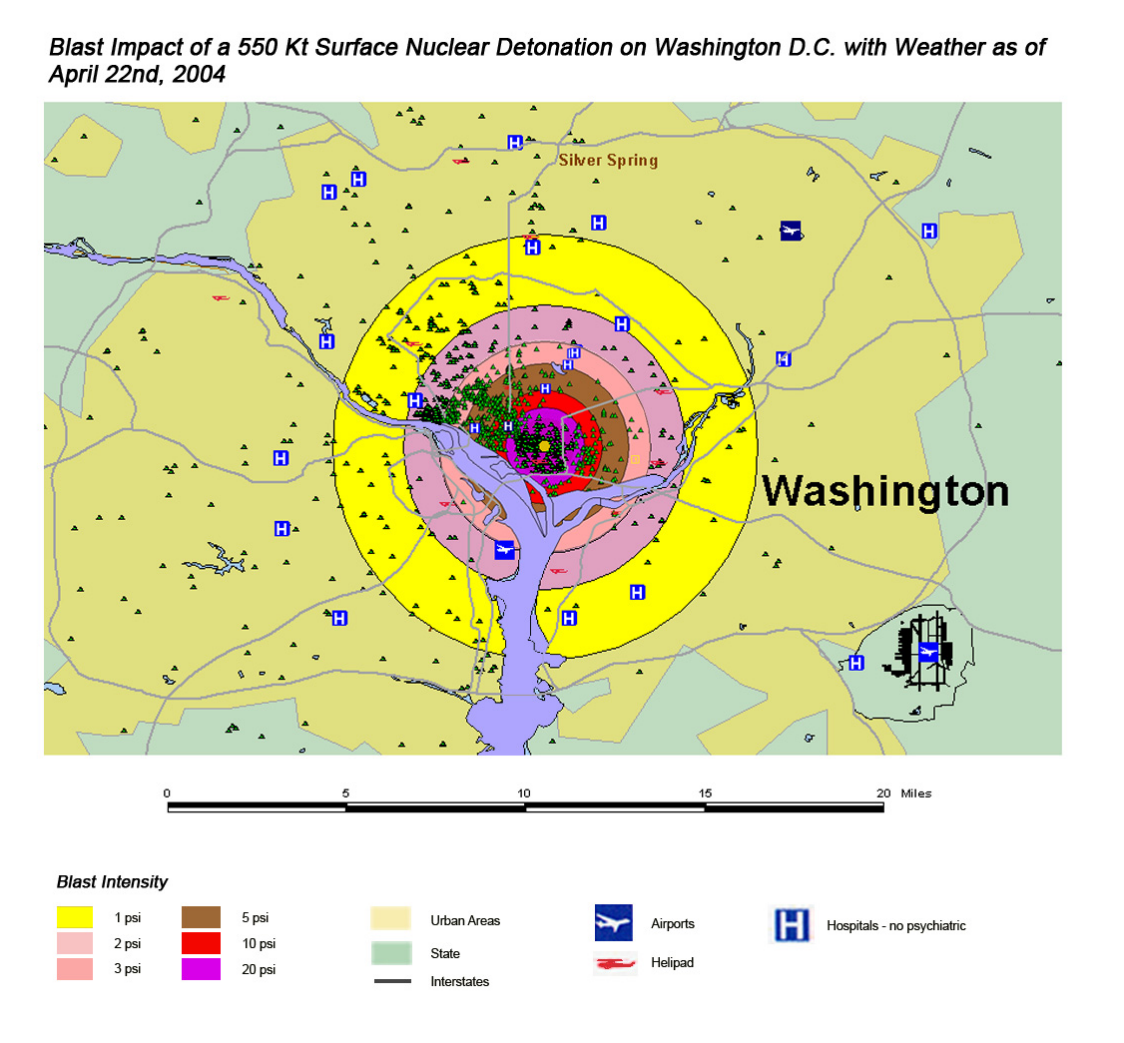
Blast Impact of a 550 Kt Surface Nuclear Detonation on Washington, D.C. with Weather as of April 22^nd^, 2004.

**Figure 9:**
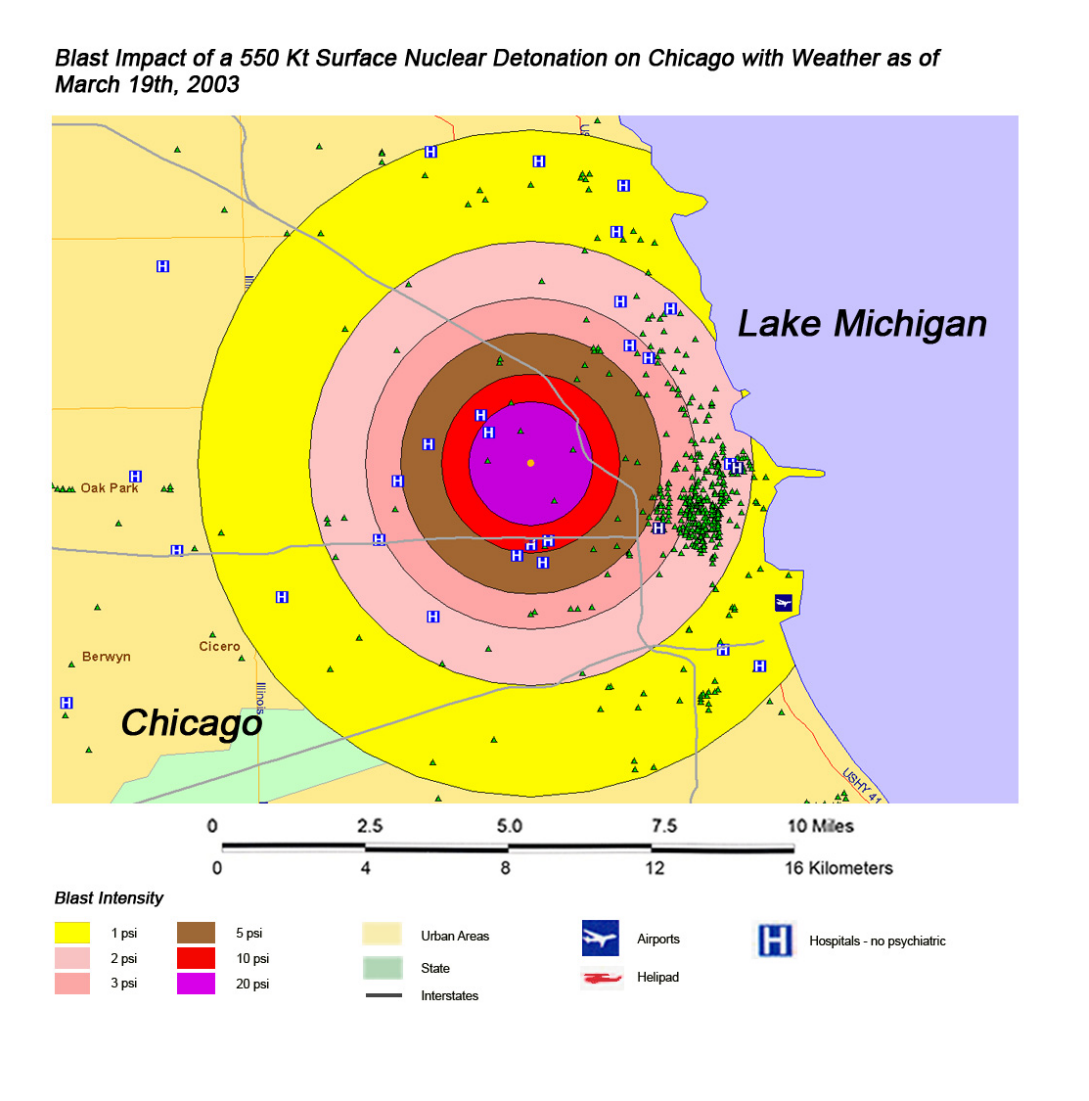
Blast Impact of a 550 Kt Surface Nuclear Detonation on Chicago with Weather as of March 19^th^, 2003.

**Figure 10:**
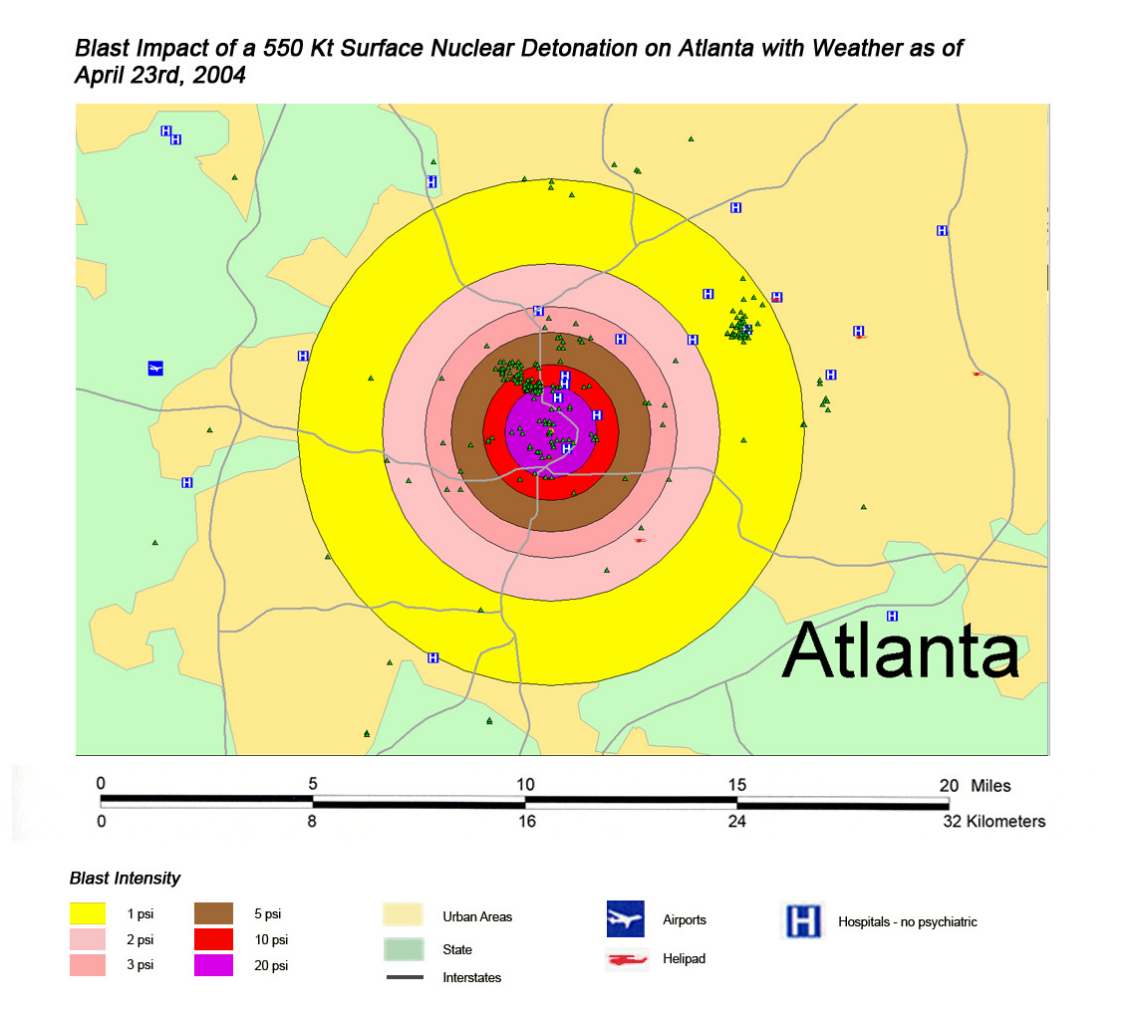
Blast Impact of a 550 Kt Surface Nuclear Detonation on Atlanta with Weather as of April 23^rd^, 2004.

The areas of New York, Washington, D.C., Chicago, and Atlanta affected by blast from a 550 Kt nuclear detonation are shown in Figures 7, 8, 9, 10, respectively. One staggering factor of the blast damage in Washington is the extremely high concentration of government buildings within the blast zones, with higher concentration of buildings corresponding to a higher degree of blast damage (Figure 7). With the overlap of blast damage with thermal effect zones, there is a similar decrease of blast damage coverage in New York due to the presence of river systems as occurred with thermal effects (Figure 8). The lack of hospitals west of the Hudson River, for instance, results in a relatively small impact of blast damage on health care systems in that approximate half of the blast zone (though the relative lack of access to health care in this area will only be exacerbated in this crisis). As noted previously with a 550 kT detonation in Chicago, location next to a large body of water helps to dissipate the effect of the blast damage over this unpopulated area (as it did for thermal effect). Indeed, for the example of this nuclear attack simulation, ground zero was placed further to the West in anticipation of this factor (Figure 9). If large-scale nuclear devices are detonated immediately adjacent to large water systems in their likely placement in downtown areas, this will consistently lower blast damage and thermal effects. The example of Atlanta, with the widespread distribution of government buildings outside the downtown urban area, demonstrates the disseminated effect in cities located in interior locations away from significant water systems (Figure 10). However, there still is a disproportionate effect on health care systems, though not as distinctive as in the other cities in this study.

#### Source Region Electro-magnetic Pulse [SREMP]

Electrical and electronic equipment, both plugged-in and some unplugged, will be severely impacted in areas affected by Source Region Electro-magnetic Pulse [SREMP]. SREMP is produced by low-altitude nuclear bursts and will affect areas from 3–8 km radius from the detonation point depending upon yield [33], with National Planning Scenarios assuming 4 kms for a 10 Kt device. This is roughly the same region likely to be affected by blast and shock. For hospitals this means power and any connected backup power sources will be lost, and most equipment connected using a plug to access power will likely have been destroyed by SREMP. Equipment that is unplugged may or may not be affected. SREMP affected areas extend up to the 1psi blast contour for small blasts (< 20 Kt) and up to somewhere between the 1 and 2psi contours for our 550 Kt example.

The combination of SREMP on electronics, and blast effects on antenna integrity and alignment will severely curtail radio, cell phone and satellite communications in a post event environment [46].

#### Prompt radiation

Prompt radiation occurs from fission products in the first second after a nuclear explosion. Significant health effects extend out to roughly 2 kms for a 20 Kt nuclear detonation and to 3–4 kms for a 550 Kt device, depending upon the radiation characteristics of the actual device. In general, radiation doses closer to ground zero are very high with a rapid fall off in dose as one proceeds outward. Within the inner zone near ground zero fatalities are generally 100% for those exposed in the open, and, even for those in buildings, mortality will be high except for those in basements.

#### Fallout radiation

The conical-shaped plumes of casualties generated by radioactive fallout account for the largest geographic distribution of effect from most nuclear weapon detonations. Most of the radioactive particles generated by the blast will fall within 24 hours on areas extending out from ground zero in the direction of prevailing winds and is referred to as early fallout.

From the radioactive fallout, the larger, relatively more radioactive particles fall out closer to the detonation area within hours. Known generally as "early fallout" this constitutes by far the greatest hazard to health. Slightly smaller particles generated by the nuclear blast will behave like aerosols and are dispersed into the troposphere where they could stay suspended for months. The fallout from this portion remains in bands around the earth at the latitude of the detonation. This portion of the fallout is often referred to as "late fallout", and is less hazardous than early fallout [47]. Additional fallout penetrates the stratosphere and its particles are deposited worldwide over a period of months to years [28]. Most of the radioactive fallout is downwind from the explosion and up to 70 per cent is in the larger particle portion, or "early fallout" occurring within hours. One principle of note is that the intensity of the radioactivity varies inversely with distance from the site of explosion. With a steady wind, the pattern of accumulated dose of radioactivity assumes the shape of nested cigar-shaped contours, each contour denoting a particular dose [48].

In a nuclear explosion, over 400 radioactive isotopes are released into the biosphere. Among these, about 40 radio nuclides are considered potentially hazardous [49]. Of particular interest are those isotopes whose organ specificity and long half-lives present a danger of irreversible damage or induction of malignant alterations [50,51]. Both early and delayed fallout result in the deposition of radioactive material in the environment [52]. The annual average whole-body fallout rate in the United States at the end of the 20^th ^Century was approximately 45 FSv (4.5 mrem) [53,54].

To consider the relative long-term impact of fallout, a device about twice the size of the 550 Kt weapon analyzed in this study (one MT), detonated at ground level with a steady wind of approximately 15 miles per hour, would produce a fallout radioactivity dose rate of 400 rem in 24 hours in an area of approximately 400 square miles. At a dose rate of 2 rem per year, more than 20 times the maximum recommended by the EPA, an area of 1,200 square miles would remain unfit for use for a year and more than 20,000 square miles would be uninhabitable for a month [55].

Several Federal Web sites offer good discussions of nuclear issues including fallout [56-58]. The Department of Homeland Security has a number of ongoing initiatives such as the Radiological and Nuclear Countermeasures Program to enhance U.S. security against unconventional attacks. Their summary provides an excellent background on where we are today and where we are going, as well as some useful theory [59]. Should a real event occur, federal assistance can be provided by specialized teams, such as the Oak Ridge Institute for Science and Education's (ORISE) Radiation Emergency Assistance Center (REAC/TS) [60]. These teams can also provide pre-event nuclear and radiation training.

**Figure 11:**
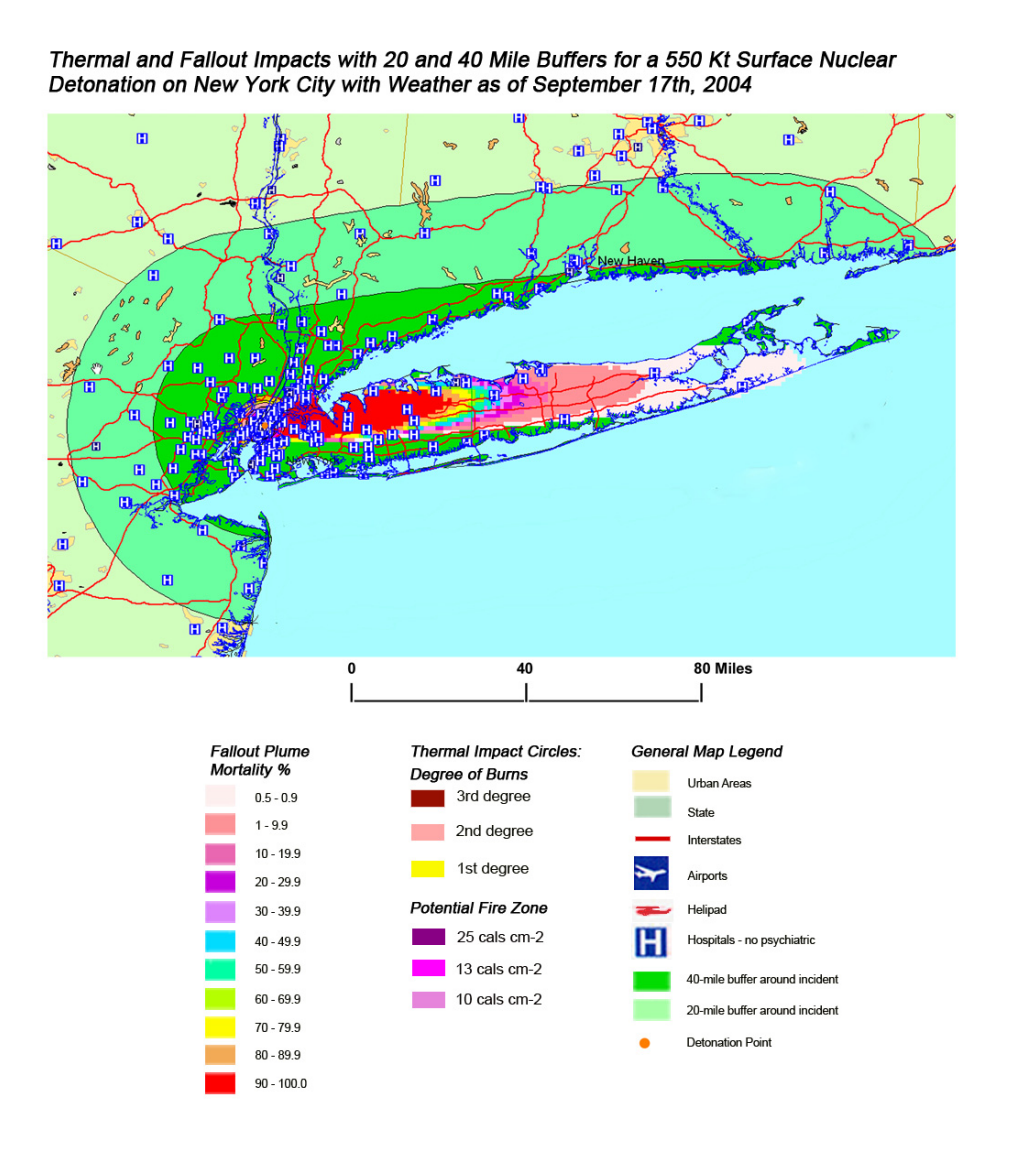
Thermal and Fallout Impacts of 550 Kt Surface Nuclear Detonation on New York City with Weather as of September 17^th^, 2004.

**Figure 12:**
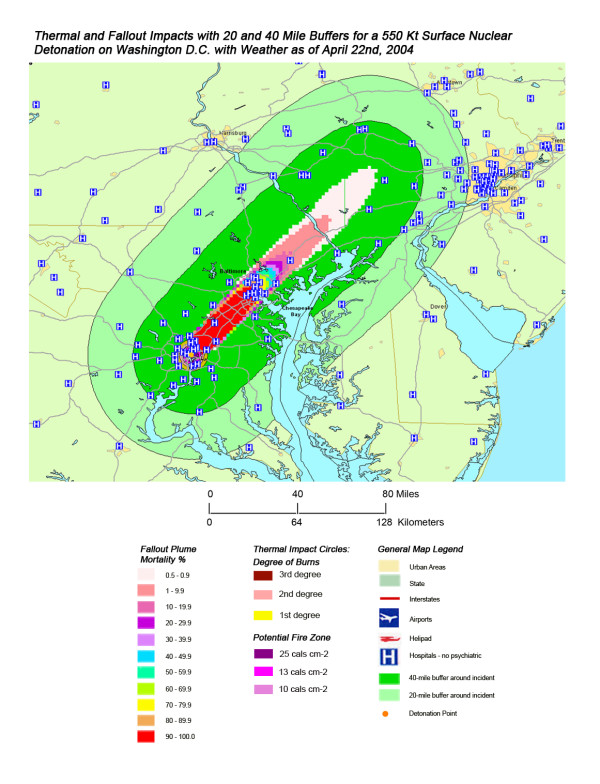
Thermal and Fallout Impacts of a 550 Kt Surface Nuclear Detonation on Washington, D.C. with Weather as of April 22^nd^, 2004.

**Figure 13:**
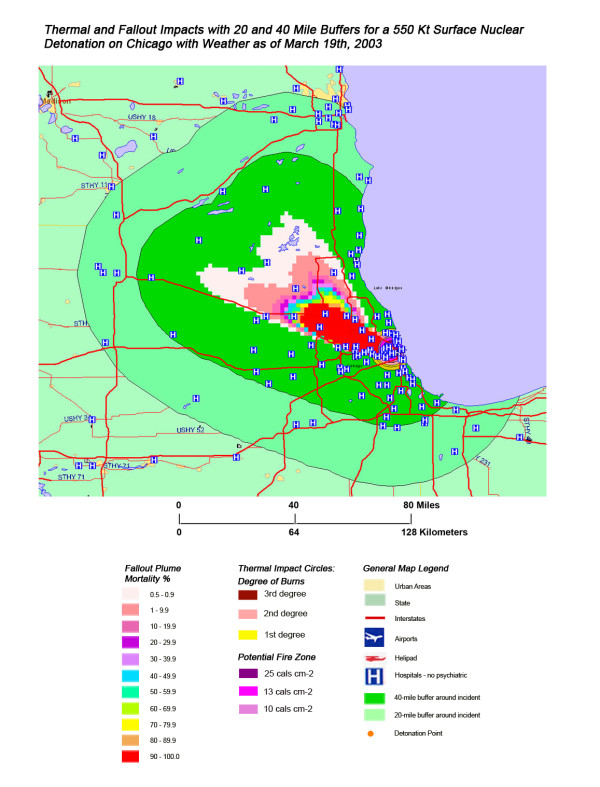
Thermal and Fallout Impacts of a 550 Kt Surface Nuclear Detonation on Chicago with Weather as of March 19^th^, 2003.

**Figure 14:**
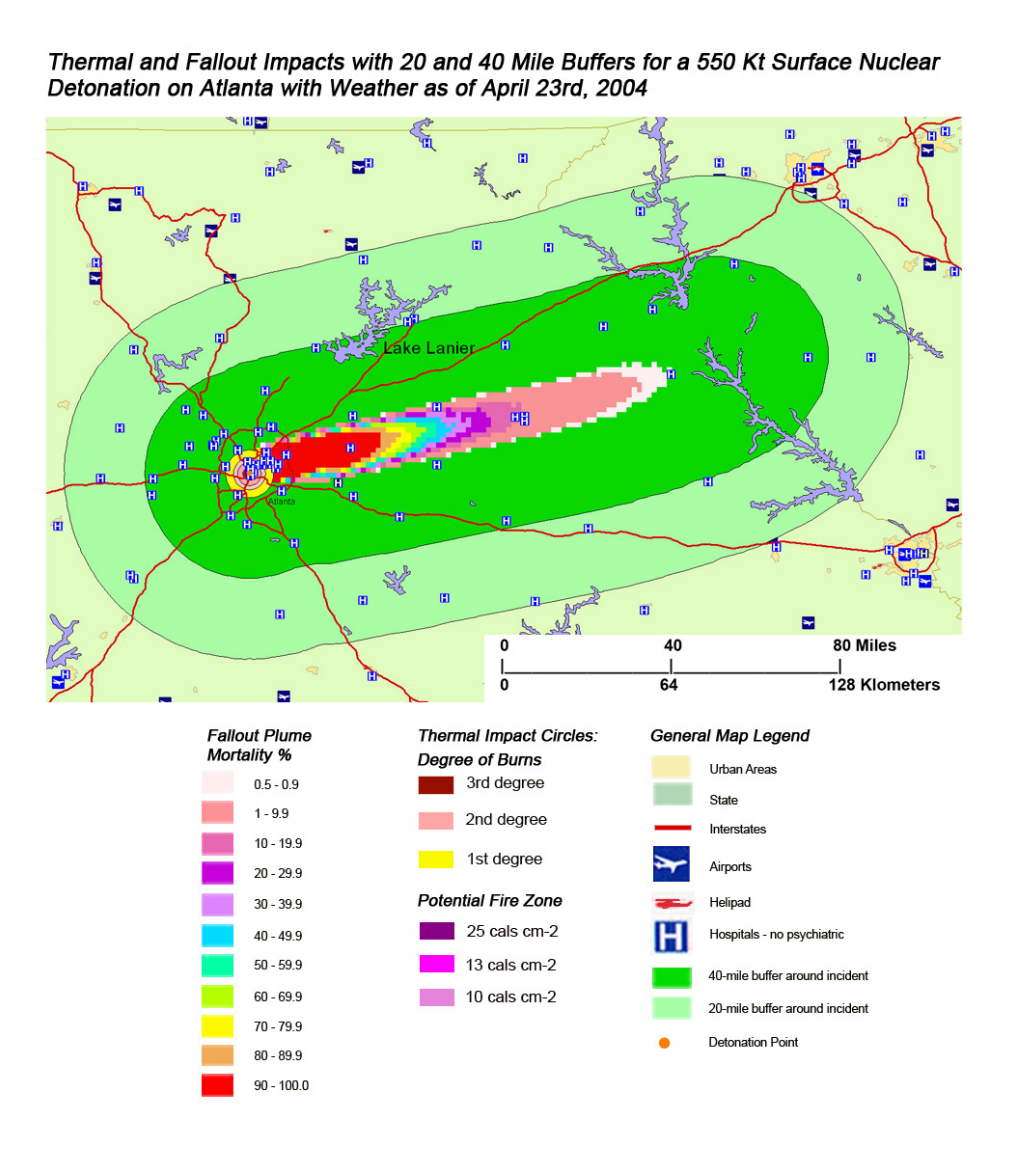
Thermal and Fallout Impacts of a 550 Kt Surface Nuclear Detonation on Atlanta with Weather as of April 23^rd^, 2004.

**Figure 15:**
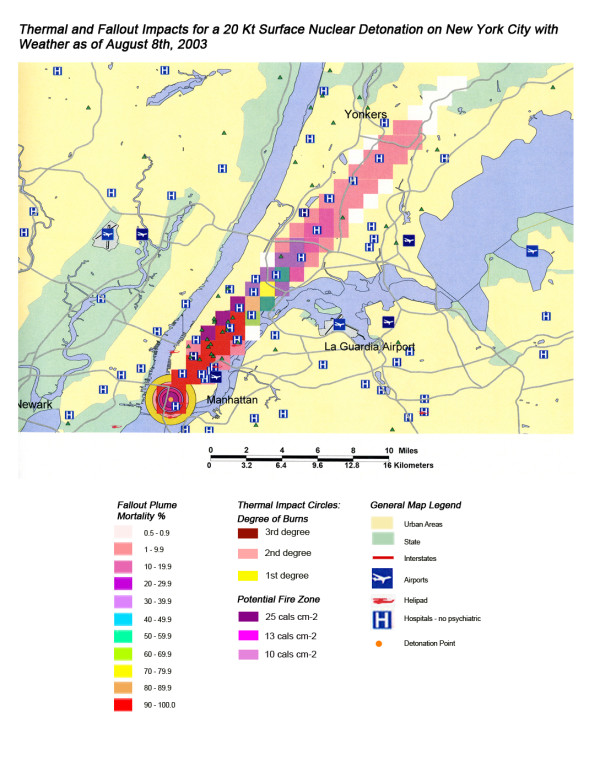
Thermal and Fallout Impacts of a 20 Kt Surface Nuclear Detonation on New York City with Weather as of August 8^th^, 2003.

**Figure 16:**
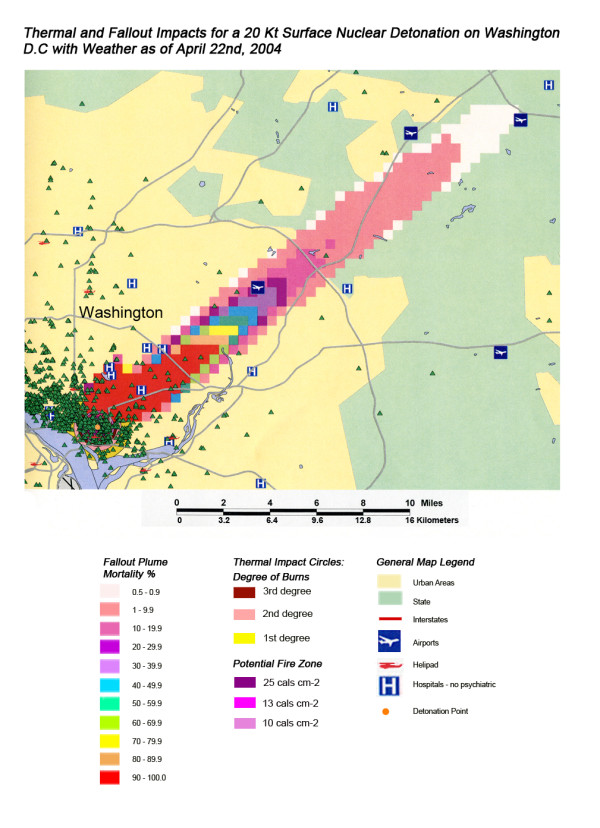
Thermal and Fallout Impacts of a 20 Kt Surface Nuclear Detonation on Washington, D.C. with Weather as of April 22^nd^, 2004.

**Figure 17:**
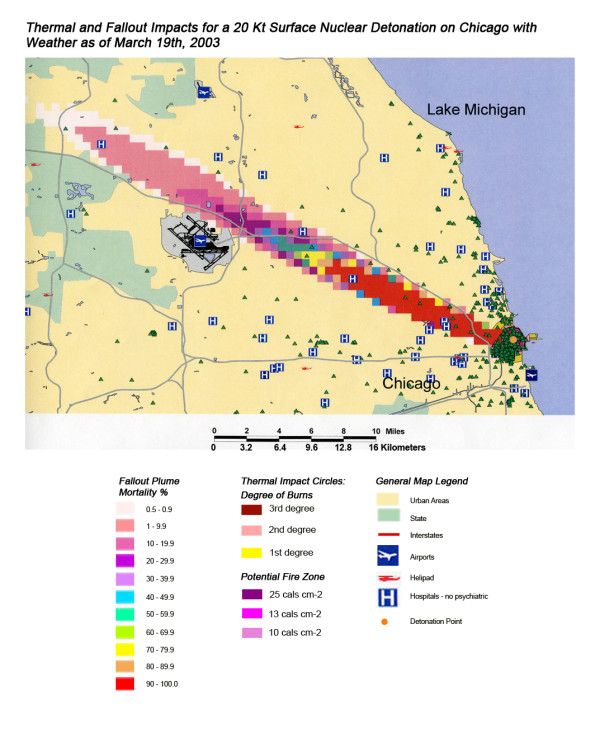
Thermal and Fallout Impacts of a 20 Kt Surface Nuclear Detonation on Chicago with Weather as of March 19^th^, 2003.

**Figure 18:**
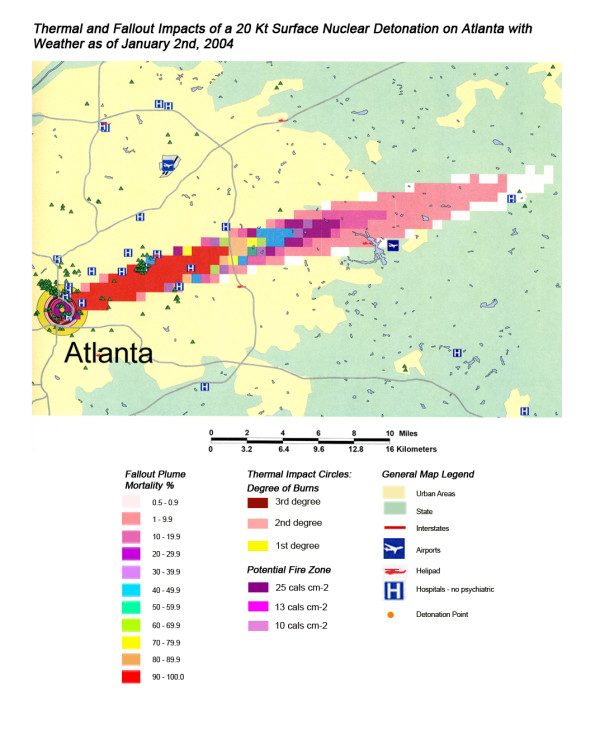
Thermal and Fallout Impacts of a 20 Kt Surface Nuclear Detonation on Atlanta with Weather as of January 2nd, 2004.

The areas of New York, Washington, D.C., Chicago, and Atlanta affected by fallout and thermal from 550 Kt and 20 Kt nuclear detonations are shown in Figures 11, 12, 13, 14 and Figures 15, 16, 17, 18, respectively. In the case of New York, the prevailing West to East weather pattern results in a conical extension of fallout casualties down the length of Long Island following the 550 kT detonation in Manhattan (Figure 11). This scenario carries significant negative impacts on the health care systems distributed consistently along the length of the island, with 51% of hospitals and 53% of the medical staff lost within 20 miles of ground zero (Table 4). This is the highest number of affected hospitals (at 54) in this publication, for all four cities considered. Even for the smaller 20 Kt weapon in New York (Figure 15), wind patterns coming inland off of the ocean result in a devastating loss of the great majority of health care systems located between the East and Hudson Rivers due to the resulting fallout.

**Table 4:** Effect of 550 Kt detonation on health care in four cities*

**Affects of 550 Kt Detonation within 0.1% mortality contour and 1psi**	Washington	New York	Chicago	Atlanta
Hospital Beds in Affected Area (in thousands)	9.9	24.5	12.1	4.5
Total Beds within 20/40 miles of incident edge (in thousands)	7.3/13.1	24.0/33.6	9.8/17.3	4.4/10.3
%Beds lost within 20/40 miles of incident edge	57/43	51/42	55/41	50/43
Number of Hospitals in Affected Areas	30	54	42	14
Additional Hospitals within 20/40 miles outside affected area	33/61	77/107	45/72	23/40
% Hospitals lost to incident	48/33	41/34	48/37	38/26
Number of Health Staff Affected by incident (in thousands)	67.1	145.3	75.1	28.8
Additional Health Staff within 20/40 miles (in thousands)	41.7/81.0	127.4/178.2	58.0/99. 8	25.8/35.2
% Health Staff Lost	62/45	53/45	56/43	53/45

• Effect contour was considered to be 0.1% mortality for radiation or 1 pound per square inch for blast, which would break windows and also subject staff to some burn effects when exposed to thermal radiation.

The stunning impact of fallout radiation from the 550 Kt detonation is evident from the loss of the hospital systems from two major metropolitan areas: the loss of Washington D.C. health care systems from the thermal and blast effects and the loss of Baltimore hospitals from the fallout plume 40 miles away (Figure 12). This resulted in a 48% loss of hospitals in the 20 mile buffer around the two cities, a 57% loss of beds, and 67,000 health care workers directly affected for a total loss of 62% of the workers (Table 4). In Figure 16, the mass fire zones in Washington D.C. after a 20 Kt detonation are hardly visible due to the large number of government buildings in the burned out areas. Even for this relatively small nuclear device, half of the hospitals in the immediate vicinity of the city will be circumscribed by the fallout plume.

Not all fallout plumes will be symmetrical cones in shape, as demonstrated in Figure 13 in Chicago, with the weather pattern for that day resulting in a broader, heart-shaped plume extending from ground zero into the interior beyond Lake Michigan. Such expansions can greatly increase the impact on health care systems, as indicated by the 48% loss of hospital beds within 20 miles of the plume area (Table 4). This is similar to the hospital bed loss rate for both cities affected by the Washington detonation (Washington D.C. and Baltimore), and considerably higher than the hospital loss rates for New York and Atlanta. The actual percentage of hospital bed loss also was higher for Washington D.C. and Chicago than for New York and Atlanta. Unlike Washington D.C. though, the smaller 20 Kt detonation did not have the same inclusive effect on hospital loss, with the narrow radiation plume in the Chicago example leaving a large number of unaffected suburban hospitals intact. In this case, the significant urban sprawl of America's third largest city has resulted in a sufficiently widespread distribution of suburban hospitals, resulting in a significant number of hospitals escaping deactivation from the relatively narrow fallout plume. A similar situation is seen with Atlanta (Figure 18), where the narrow fallout plume of the smaller 20 Kt device devastates several key urban hospitals, but many more in the suburban sprawl are apparently spared contamination resulting in immediate threat to life.

### Measuring radiation dosage

Like most drugs or chemicals, there is a relationship between radiation dose and its effect on the body. Radiation dosing can be thought of as an amount of energy absorbed by the body. The rad is a unit of absorbed radiaton dose defined in terms of the energy actually deposited in the tissue. One rad is an absorbed dose of 0.01 joules of energy per kilogram of tissue. To accurately assess the risk of radiation, the absorbed dose energy in rad is multiplied by the relative biological effectiveness (RBE) of the radiation to get the biological dose equivalent in rems. The RBE is a "quality factor," often denoted by the letter *Q*, which assesses the damage to tissue caused by a particular type and energy of radiation. For alpha particles, *Q *may be as high as 20, so that one rad of alpha radiation is equivalent to 20 rem. The *Q *of neutron radiation depends on their energy. However, for beta particles, x-rays, and gamma rays, *Q *is taken as one, so that the rad and rem are equivalent for those radiation sources [61,62].

### Overall effects

The effects of thermal, blast and radiation for both 20 and 550 Kt events can be readily seen in figures 2a through 2f. Blast and thermal effects can be compared for 20 and 550 Kt detonations in figures 2a and 2b, and 2c and 2d respectively. All four of the figures are on the same scale. The figures readily bring out the relative importance of blast compared to thermal for the smaller 20 Kt event (compare figure 2(a) to 2(c)) and the much larger effects of thermal with the larger 550 Kt event (compare figure 2(d) to 2(b). The much greater relative size of the fallout plume for a 550 Kt compared to a 20 Kt event is easily seen in figures 2e and 2f, which are at the same scale.

Despite the smaller effect of thermal compared to blast for the 20 Kt detonation, it must be emphasized that hospitals have very few burn beds in the entire U.S.A. (< 1500) and only a few (less than 150) are not occupied at any one time. Even a small nuclear event will totally overwhelm our hospitals' ability to take care of resulting burn casualties.

### Effects of 20 Kt surface detonation

In the first 750 m (12psi) virtually all buildings will be destroyed by blast, mass fires are common and prompt radiation doses are fatal except in basements, resulting in very few survivors. Between 750 and 1250 m the peak overpressure decreases from 12 to 5psi with walls blown out of buildings, though building frames may well survive. Debris will be tens of feet thick in most downtown areas with ten-plus story buildings [45]. Roughly half of the population in this area will be fatalities, mainly from collapsing buildings, with the other half injured. Most of those surviving will have been exposed to a fatal dose of prompt radiation, though death will occur first due to mass fires or third degree burns. Between 1250 m and 1750 m peak overpressures will fall from 5psi to near 3psi, and burn thresholds towards the edge of this zone will drop from third degree to second degree levels. Prompt radiation also will typically drop quickly from over 800 rem at 1600 m to over 400 rem at 1700 m. At 1900 m or 3psi, large numbers of trauma injury would ensue from walls blown out of steel framed buildings, severe residential damage and people caught in the open. By 2000 m, burn risk will drop to first degree levels. At up to 3800 m or 1 psi people will be endangered with flying glass and debris from damaged structures and glass will break out to over 6 kms, exposing those houses in the downwind fallout radiation zone to more radiation.

### Effects of 550 Kt surface detonation

A 550 Kt detonation differs dramatically from a 20 Kt one as the thermal effect increases dramatically in proportion to blast effects. Thermal intensities of 25 cals/cm2 with high mass fire probability reach out 4.7 kms or as far as the 3.8 psi blast contour, while 13 cal/cm2 intensities with many fires or even mass fires in some cities reach out to 6.3 kms, roughly the same as the 2.5 psi blast contour.

In the first 1800 m peak overpressures will exceed 20psi, destroying even the largest and best built structures. At 2200 m overpressures will be above 12 psi, destroying virtually all but a very few specially constructed buildings. The five psi contour extends out to 3,800 m, where the walls of most buildings are blown out. Most or all of this area will have very high prompt radiation values depending upon the bomb design. In addition the area will almost certainly be consumed by fire as thermal fluences exceed 25 cals/cm^2 ^out to 4700 m. The high blast, high radiation and high thermal combination means there will be virtually no long-term survivors from these areas. High thermal fluences continue out to beyond 6 kms (13 cal/cm2) with overpressures of 2.5 psi. At 7 kms thermal fluences still exceed 10 cals/cm2, associated with 2 psi overpressures. Some of those areas will experience fires, even mass fires in some cities like Atlanta where there are many frame houses within these contours. Secondary fires will also start from damaged gas and power lines. Third degree burns extend out to 7.3 kms with 2 psi overpressure present, and second degree burns occur out to 8.7 kms with overpressures exceeding 1.5 psi. Overpressures of 1 psi with first degree burns extend to almost 12 kms and the 0.5 psi glass breakage extends out to almost 20 kms.

### Populations affected by the detonations

Tables 2 and 3 show the affected populations in each of the four cities for 550 and 20 Kt detonations. They are broken down into those affected by thermal, fallout radiation, blast hazards and combined effects. The actual percentage of the affected population in each group that becomes a casualty depends on many factors, as well as the interaction of the hazards.

### Casualties from fallout

In the most extreme example of this effect in these simulations, mortality rates from 90+% in Brooklyn extend continually across the length of Long Island to 1% at the eastern tip, with deaths due solely to radioactive fallout (without thermal or blast injuries) from the 550 Kt detonation in New York [Figure 11]. This could result in over 5,000,000 deaths in the > 90% plume area, which would extend from Brooklyn to almost half the length of Long Island (the most populous half). As evaluated in the combined injuries section, these numbers inevitably include some thermal and blast initiated deaths.

An interesting pattern in these graduated mortality plumes is that for the smaller 20 Kt detonation, the number of victims in the 50–90% radioactive fallout plumes is considerably less than in the 10–50% mortality range plumes. For example, in the 20 Kt blast for New York (Figure 15 and Table 3), for example, those mortality numbers are 145,123 and 358,922, respectively, or an approximate 1:2 ratio. This was also the case for the 20 Kt Washington D.C.(Figure 16) and Chicago (Figure 17) mortality plumes for radioactive fallout in the 50–90% and 10–50% mortality ranges. For Atlanta (Figure 18), the same pattern was also seen except the difference was even higher at almost 1:4. This higher number of fatalities in the areas with a lower percentage of mortality is due to the smaller area covered by the 50–90% mortality plumes relative to the higher (90+%) and lower (10–50%) ranges upwind and downwind. Apparently, the very high deposition rates of radioactive particles that occur in the first kilometers from the detonation rapidly drops off in magnitude with wind dispersion, with the broader areas of dispersion at the lower concentrations of radioactivity sufficient to account for remarkably higher rates of mortality.

### Combined injuries

The coincidence of the thermal and blast casualty areas emanating from ground zero generates both a zone of dual casualty categories as well as a greatly enhanced mortality and morbidity rate for the geographically impacted areas. Figures 2b and 2d show substantial areas affected by both blast and thermal hazards. The casualty model predicts a zone of mass fires with > 25 cals cm^2 ^for both sizes of nuclear weapons detonations in these simulations. In this area, the fireball generated by the blast, as well as spontaneous incineration of buildings from radiant heat, will generate mass fires that would consume the great majority of the structures above ground. This quantity of thermal energy would be expected to result in virtually complete mortality from thermal injuries alone. For all four cities, for a 550 Kt detonation the model generates a nearly equivalent geographic area for this central > 25 cals cm^2 ^mass fire zone as it does for the blast ring incorporating trauma injuries resulting from up to 3 lbs/in^2 ^blast pressure. The central blast rings of 20 and 10 lbs/in^2 ^would be expected to result in primarily complete mortality from the blast effects, like the total mortality resulting from thermal effects in the > 25 cals cm^2 ^thermal zone. However, there would have been significant survival from blast trauma effects in the 3 lbs/in2 blast ring were it not for its coincidence with the > 25 cals cm^2 ^thermal zone. These people surviving blast trauma injury would succumb instead to death from the mass fires.

Overall, the total number of affected population by thermal injuries is 30% greater than that for blast injuries for all four cities for the 550 Kt detonation predictions (Table 2). Outside the mass fire areas, there will be geographic areas dominated by either first, second, or third degree burns in surviving victims of the nuclear detonation. These three thermal injury category zones coincide approximately with the 1 and 2 lbs/in^2 ^blast ring areas in all four cities for the 550 Kt detonation simulations (compare Figures 3, 4, 5, 6 for thermal with Figures 7, 8, 9, 10 for blast). The total number of thermal injuries from the 2^nd ^and 3^rd ^degree burn areas is consistently smaller than the trauma injuries in these geographically similar areas for the 1–2 psi blast rings. For Washington, New York, Chicago, and Atlanta (550 Kt simulations in Table 2), these two burn areas together produce 73%, 75%, 72%, and 71%, respectively, of the blast injuries from 1–2 psi that occur in the same approximate area.

A comparison/contrast of blast, thermal and prompt radiation effects is best made with the 20 Kt detonation, as the dominance of thermal effects at the larger nuclear detonations masks their associations. For a 20 Kt detonation mass fires from 13 cals/cm^2 ^thermal fluences would normally extend out to a location where blast effects at the 7psi level are also present. Blast effects alone at 7psi would account for at least 10% fatalities with virtually all of the rest injured due to catastrophic structural damage and impaction or injuries from flying glass. Just 80 m closer to ground zero, fatalities due to blast at 8psi would leap to 50%. Much of this zone would likely be consumed by mass fires as it is within the 13 calorie/cm ^2^contour. It is also well within the area of intense prompt radiation with values well over 1000 rem; only those in well protected basements or in subways would escape this prompt radiation.

The third degree burn zone would extend out to where blast intensities of just over 4psi were experienced, causing major structural damage to frame houses and lighter commercial construction. In addition to burns, many injuries will occur because of movement of interior walls and objects, and impaction of humans, especially those standing, on fixed items. In addition, prompt radiation of over 1000 rem in the open will have affected the entire area, greatly compounding the recovery process for those experiencing good protection by buildings and causing death to those exposed in the open or in many types of buildings [[26], 1–10]. In this third degree burn zone, 15% of the affected population who are outside or near line-of-sight windows will die because of second degree or worse burns to their bodies followed by shock; another 40% will have buildings or walls fall on them and be killed, trapped or injured with trauma events. At least 15% will receive lethal prompt radiation doses and 10% will die in the plume from exposure to very high levels of fallout radiation. Of the 20% left, about a third will have received about 500 rem (assuming an average protection factor of 0.5 for prompt neutrons) which will eventually prove fatal in this environment. Another third will receive about 300 rem, which will prove fatal for 5–10% of them after 60 days. In the end, there may only be a 10% survival rate in the third degree burn zone (1500 m)

As we move from the 1500 meter to the 2000 meter distance from ground zero, conditions for survival improve rapidly. Peak overpressures decrease from 4 psi to just over 2.5 psi and burn injuries decrease to first degree. Two psi is reached at 2300 m and one psi at 3800 m. Prompt radiation falls off precipitously between 1600 (1,000+ rem), 1700 (400 rem) and 2000 m (80 rem). Many of those in the open will have been subject to fatal doses, but those inside with reasonable protection factors should be safe from prompt radiation in the outer parts of this zone. Mortality and morbidity will remain high for those in the fallout plume as these people will have been exposed to very high levels of radiation, with some additional blast and burn injury combinations.

Injuries from breaking glass will occur at over 6 kms, where radiation in the fallout plume is 1800 rem. Most injuries beyond 2000 m will occur due to people being caught in the fallout plume where radiation exposures, even with protection, remain in the fatal range (2400 rem in the open at 3800 m)

Due to the combination of injury categories, death rates can be exacerbated far beyond that expected for any one of the injuries taken alone. Victims cannot move and could be consumed by fire or are simply left to die due to lack of resources. Others fall victim to poor sanitation due to failure of the main power, water and waste facilities. Lack of immediate (12 hours) or even intermediate (48 hours) health care often results in the body going into shock or succumbing to infection, which would not have occurred had basic health care been available.

### Immediate deterioration of urban institutional health care resources

The nationwide trend of locating a majority of the major urban health care institutions in downtown areas would result in a staggering loss of the total institutional health care delivery following nuclear weapon use. Data is shown for the four example cities in Table 4, though we have seen very similar results in the 20 largest U.S. urban areas (data not shown).

The four cities in Table 4 show 50–56% loss of hospital beds within a 20 mile radius of a 550 Kt detonation, and a 41–43% loss of beds at a 40 mile radius from a downtown ground zero. These results are strikingly similar in view of the very different geographic and demographic landscapes of these four cities. When considering the actual number of hospitals lost, Washington D.C., New York, and Chicago are similar in magnitude of the percentage of hospitals lost, between 41–48% within 20 miles, and 33–37% lost within 40 miles of the detonation. Atlanta, which is the smallest city of the test sample, had a smaller percentage of hospitals lost compared to the others. Due to the pattern of having the larger hospitals in the downtown area, Atlanta still had a similar percentage of bed loss, even though the number of hospitals lost overall was smaller.

A closer look at the New York map (Figure 11) shows that the situation is much worse for Long Island residents as half of the hospitals within 20 miles are either west of the Hudson in New Jersey and inaccessible due to high radiation levels and/or fires on Manhattan or only accessible by water across Long Island Sound. The loss of critical tunnels and bridges from Manhattan and contamination of boats along southern Long Island Sound would vastly complicate the relief effort and medical response for Long Island residents.

As emergency planners begin to understand the importance of providing surge capacity some ameliorating events are occurring. In the State of Georgia, the Division of Public Health has purchased 11,000 portable emergency hospital beds which is an impressive increase of almost 70% over existing bed capacity for the State. These resources will be distributed around the State and, in an emergency, could be moved closer to the disaster for greater efficiency in treatment and as a means to increase the capacity of surviving hospitals. This approach is now being pursued by other states.

Obviously, the most important resource in medical response are the trained health care personnel, and it is in this area that the most dramatic impact of a nuclear detonation is seen on overall health care response. Losing at least half of your health care responders in the first minute of the attack is all the more damaging because so many of the thermal and trauma injuries require immediate care and cannot wait for the time-consuming importation of replacement medical workers.

Another issue deals with medical and credentialing records. Currently many records are stored in inner city areas and may be lost in an attack. Many hospital records are not stored off site and patient and staff records could be lost or made inaccessible. Much work needs to be done on supporting informatics to ensure overall post-event success.

One very important finding in the loss of hospitals and medical resources from urban nuclear attack is the potential for a relatively greater impact of thermal injuries versus blast effects as the magnitude of the nuclear device increases. Comparison of Figures 3 and 7, representing thermal and blast impacts, respectively, for a 550 Kt detonation in New York, demonstrates that overall there is a greater radius of impacted hospitals from thermal effects than blast effects. This pattern is repeated for Washington D.C. (Figures 4 &8), Chicago (Figures 5 &9), and Atlanta (Figures 6 &10). The outer edges of effects for the blast effects, with 1 or 2 psi, could be expected to impact the hospitals in those areas certainly, by blowing out windows, moving equipment around, and, in combination with thermal effects, injuring 25–45% of the population. However, the outer edge of the second degree burn zone burns extends roughly to the 1.5 psi radius (5.4 miles) and would severely impact personnel in any hospital in direct line of sight to the explosion. First degree burn effects extend out further (7 miles), roughly to the 1 psi contour. Even if some attempt to restore the hospital infrastructure was made, combined thermal, EMP and blast injuries would make it unlikely that the personnel would be able to function effectively, especially in a mass casualty crisis.

### Future directions for improvement in casualty models to expedite disaster response

One of the largest limiting factors of these models is that they require one to model an event of a known size. Initial data will be inadequate to estimate the size of the weapon with any certainty. Is it a 5, 10, 20 Kt or larger event? As more information becomes available, better estimates may be made. The collection of relevant real time data from field sensors would greatly improve early estimates of the event size and event impact, enable the models to be run iteratively, making their output more reliable with time and greatly improve decision-making. To maximize the efficacy of these models and their associated databases, responders, from the emergency medical technician on scene, all the way up to the incident commander, must understand in general terms the capabilities and limitations of our models. Accordingly, it would be necessary to involve them in tabletop and field exercises involving the use of models.

Improved calculation of thermal effects (including burns and mass fires) and fallout estimates for dense multi-storey urban environments in major US cities require more detailed databases. DTRA is already coordinating the creation of building databases for priority U.S. cities. Oak Ridge National Laboratories is preparing a more accurate LandScan USA database on a 90 meter grid with both daytime and nighttime population estimates for future release [63]. Detailed land use and tax parcel data and building information (height, construction date and type, number of stories, etc) are being acquired for several cities and will help to further refine the models and test the sensitivity of the casualty estimates to different variables. The incorporation of numbers of people actually present in downtown and in the suburbs during working hours will improve our predictions immensely The addition of detailed journey-to-work (origin-destination) data and building-level population data from fire departments and insurance risk assessments to estimate daytime populations in urban centers will make for better casualty planning and management

These increased capabilities allow detailed Geographic Information Systems analyses of the impact of potential mass fires, and, of first, second and third degree burns, and fallout and blast from nuclear incidents. Analysis of block group data allows the impact of skin color and age to be taken into account for better estimating burn intensity and fallout mortality. More detailed data on buildings allow the use of better radiation protection factors, which improves casualty estimations further. They also improve life and death decision-making processes such as shelter-in-place or flee. These additional data permit the interaction of blast, thermal and fallout effects to be better modeled and thus generate more robust estimates of different types of potential mass casualties which, in turn, help us plan better responses for casualty prioritization and treatment, given the limited medical resources that will be available in the first few days after an incident.

While preparing for the potential use of WMD within areas that have not seen mass casualties previously (such as the United States) is of critical importance, these "upgrades" of emergency response capabilities will also have important "peacetime" benefits. Geographic information systems used in tracking releases of toxic chemical and radioactive agents and mobilizing emergency response resources to targeted areas would also be highly useful in responding to tornado and flood disasters. While we can continue to hope that large-scale mass casualties from WMD attacks will remain high consequence, low probability scenarios, it is mandatory that we invest the appropriate physical and human resources to deal with such a staggering prospect.

## Competing interests

The author(s) declare that they have no competing interests.

## Authors' contributions

WCB and CED jointly conceived the paper concept, formulated the focus on health care vulnerability, and selected the scenarios from a much larger available set of urban nuclear attack simulations conducted by this group. WCB programmed the thermal and blast effects, built the database and ran the GIS and CATS/HPAC models. CED and WCB jointly analyzed the data outputs and shared in the manuscript composition. Both authors read and approved the final manuscript.
